# Specifically bound BZIP transcription factors modulate DNA supercoiling transitions

**DOI:** 10.1038/s41598-020-75711-4

**Published:** 2020-11-02

**Authors:** Johanna Hörberg, Anna Reymer

**Affiliations:** Department of Chemistry and Molecular Biology, University of Gothenburg, 40530 Gothenburg, Sweden

**Keywords:** Biophysical chemistry, DNA, DNA-binding proteins, Molecular modelling, Computational chemistry, Molecular dynamics

## Abstract

Torsional stress on DNA, introduced by molecular motors, constitutes an important regulatory mechanism of transcriptional control. Torsional stress can modulate specific binding of transcription factors to DNA and introduce local conformational changes that facilitate the opening of promoters and nucleosome remodelling. Using all-atom microsecond scale molecular dynamics simulations together with a torsional restraint that controls the total twist of a DNA fragment, we address the impact of torsional stress on DNA complexation with a human BZIP transcription factor, MafB. We gradually over- and underwind DNA alone and in complex with MafB by 0.5° per dinucleotide step, starting from the relaxed state to a maximum of 5° per dinucleotide step, monitoring the evolution of the protein-DNA contacts at different degrees of torsional strain. Our computations show that MafB changes the DNA sequence-specific response to torsional stress. The dinucleotide steps that are susceptible to absorbing most of the torsional stress become more torsionally rigid, as they are involved in protein-DNA contacts. Also, the protein undergoes substantial conformational changes to follow the stress-induced DNA deformation, but mostly maintains the specific contacts with DNA. This results in a significant asymmetric increase of free energy of DNA twisting transitions, relative to free DNA, where overtwisting is more energetically unfavourable. Our data suggest that specifically bound BZIP factors could act as torsional stress insulators, modulating the propagation of torsional stress along the chromatin fibre, which might promote cooperative binding of collaborative DNA-binding factors.

## Introduction

Torsional restraints on DNA, referred to as DNA supercoiling, constantly change during the life of the cell, and regulate transcriptional control on many levels^1–5^. DNA supercoiling represents a sum of writhe and twist—the two interchangeable variables. DNA writhing generally dominates supercoiling changes on a larger scale through the formation of loops and knots, while DNA twisting occurs when shorter DNA fragments, up to ~ 100 base pairs (b.p.), experience changes in torsional restraints. The net state of genomic DNA is neutral, but regions of positive and negative supercoiling can exist locally, created by RNA polymerases that expose DNA to torsional stress^3,5^. This introduces DNA undertwisting (negative supercoiling) upstream and overtwisting (positive supercoiling) downstream of a transcribed gene.

Torsional stress can propagate along DNA, modulating transcription of near-located genes^1,5^ by altering the stability of nucleosomes and other protein-DNA complexes^3,4,6,7^, changing the accessibility of the genetic code. The ranges and speeds of torsional stress propagation depend on the underlying nucleotide sequence^1^. Computational experiments confirm: DNA responds to torsional stress in a heterogeneous and sequence-dependent manner^8–10^. Certain dinucleotide steps, mainly pyrimidine-purine (YpR) but also purine-purine (RpR), in specific sequence environments, absorb a large part of DNA over- and undertwisting, while the rest of the molecule preserves its relaxed B-like conformation. The torsional plasticity of these dinucleotides is founded in the polymorphic nature of the DNA backbone^11–13^. When absorbing torsional stress, these dinucleotide steps favour respectively low (DNA-underwinding) or high (DNA-overwinding) twist states, which are separated by about 20°. The twist transitions are coupled with significant changes in other helical parameters, such as shift and slide. We hypothesize that these dinucleotide steps are potential 'hot spots' for transcriptional control, as they can regulate supercoiling transitions, the deformability of DNA, and specific binding by transcription factors.

Transcription factors (TFs), while operating in the large excess of non-specific DNA, must unmistakably bind their corresponding DNA targets to correctly initiate transcription events. The specific binding of TFs is usually considered in terms of three mechanisms. (1) The ‘direct readout’ which involves the formation of specific hydrogen bonds and hydrophobic interactions between DNA bases and protein residues side chains^14,15^. (2) The ‘indirect readout’ that involves recognition of the DNA shape^16,17^. (3) The water-mediated interactions between DNA and protein^18,19^. The three recognition modes can contribute differently to the specificity of TF-DNA binding, depending on the type of TF and the recognized DNA sequence. Irrespective of the dominating recognition mechanism, torsional stress passing through the genome will change the geometry of the DNA helix, and potentially alter the stability of a TF-DNA complex. Presence of a protein will likely affect the free energy of DNA torsional stress propagation, potentially regulating transcription of nearby genes. Despite being central for eukaryotic transcriptional control, these mechanistic aspects of TFs-DNA interactions are far from being understood in detail.

Hence, motivated by the scarcity of mechanistic studies, we conduct a computational experiment addressing the impact of torsional stress on DNA complexation with MafB^20–22^, a member of human BZIP family of TFs. MafB forms homo- and heterodimers with other members of the BZIP family to regulate genes involved in key cellular processes such as cell development and cell differentiation^20,23^. The MafB homodimer binds to DNA containing the Maf response element (MARE), TGCTGACGTCAGCA^20^. To recognize MARE-DNA, MafB dimer utilizes the direct readout mechanism, where a six-residues motif (**R**xxx**N**xx**YA**xx**CR**) of each monomer forms specific contacts via the major groove of the MARE-half site—TGCTGAC^24^ (Figures S1 and S2). We perform extensive all-atom umbrella sampling simulations together with a torsional restraint that controls the total twist of a DNA molecule^9^, without restricting any other degrees of freedom. Using the restrain we gradually over- and underwind DNA alone and in complex with MafB until a maximum of ± 5° per base pair (b.p.) step is reached. Our computations show that MafB changes the sequence-specific response of DNA to torsional stress by making the b.p. steps that are expected to absorb the majority of the applied torsional stress rigid. This results in an asymmetric free energy profile, where overwinding becomes significantly more unfavourable. However, irrespective of the degree of torsional stress, MafB remains stably bound to DNA, and maintains most of the specific contacts with the MARE region. Our data suggest that specifically bound BZIP factors could act as torsional stress insulators, modulating the propagation of torsional stress along the chromatin fiber, which might promote cooperative binding of collaborative DNA-binding factors and regulate the firing potential of the occupied promoters.

## Methods

### Simulated systems

We study two systems: MafB-DNA complex (PDB ID: 4AUW)^20^ and free DNA in B-form, which was created using the modelling program JUMNA^25^. Both systems contain a DNA 26-mer oligomer: GGTAAT**TGCTGACGTCAGCA**TTATGG, with MARE region in bold. For each system, the twist restraint^9^ is applied to the central MARE region between b.p. 7 and b.p. 20; 14 b.p. in total. Starting from a fully relaxed state, the total twist of the MARE region is gradually increased and decreased in steps of 0.5°/b.p step (± 6.5° in total per umbrella window), until a maximum overwound and underwound state of 5°/b.p. step is reached (± 65° in total). The final structure from every window is used as the starting point for the following umbrella window, following a so-called cascade umbrella sampling scheme^26^.

### Molecular dynamics simulation protocol

Molecular dynamics (MD) simulations and cascade umbrella sampling are performed using the MD engine GROMACS v5.1^27^. In addition, the restrained MD simulations were carried out with the help of the previously developed torsional restraint^9^ that controls the total twist of a DNA fragment between b.p. 7 and 20, restrained to the desired value of *twist*_*ref*_ using a simple quadratic function, *K*_*tw*_ *(twist − twist*_*ref*_*)*^*2*^, implemented via PLUMED v2.2^28^. The force constant (*K*_*tw*_) was set to 0.06 kcal mol^−1^ degrees^−2^, the smallest value that enables the desired torsional strain without inducing any structural artefacts. All simulations are performed using a combination of AMBER 14SB^29^ and Parmbsc1^30^ force fields to treat the protein and DNA, respectively. Selected combination of the force fields has been extensively tested and has been shown to accurately reproduce a wide range of experimental data^30–32^. In particular, Parmbsc1 has shown to capture the sequence-specific conformational mechanics of DNA, which is crucial for the correct representation of DNA behaviour under torsional stress conditions; as observed experimentally in a crystal structure of torsionally-stressed 2D triangular self-assembled DNA lattice (PDB ID: 5EOS)^33^ and computationally in short linear DNA oligomers^9,10^.

The MafB-DNA complex and free DNA oligomer are separately solvated in truncated octahedron periodic boxes by SPC/E water molecules^34^ with a buffer distance of 15 Å to the walls, and subsequently neutralized by K+ counterions. Additional K+ and Cl− ions are then added to reach a physiological salt-concentration of 150 mM. Applying periodic boundary conditions, each system is subjected to energy minimization with 5000 steps of steepest descent, followed by a 500 ps equilibration-run at constant volume, while raising the temperature to 300 K. Simulations are then carried out at constant pressure and temperature (1 atm and 300 K), where the temperature is controlled by a weak-coupling thermostat^35^ with a coupling constant of 0.2 ps and the pressure is controlled by an isotropic Paririnello-Rahman barostat^36^ with a coupling constant of 2 ps. All bonds involving hydrogen atoms are constrained with the LINCS algorithm^37^, allowing to set the time step to 2 fs. Electrostatic interactions are treated with the Particle Mesh Ewald^38^ summation method using a short-range cutoff of 10 Å. The van-der-Waals forces are also truncated at 10 Å with added long-range corrections. The neighbour pair list for nonbonded interactions is updated every 20th step through the Verlet cutoff scheme^39^. Centre of mass movement is removed every 0.2 ps to eliminate translational kinetic energy^40^.

The initial 100 ns of unrestrained MD-trajectory, considered as equilibration, is followed by a production run of 0.5 μs. Following unrestrained MD simulations, the cascade umbrella sampling is performed with 0.5 μs sampling time per window to allow sufficient convergence of DNA conformational substates and ion populations^41^. The Weighted Histogram Analysis Method (WHAM)^42^, implemented in PLUMED is used to derive the potential of mean force (PMF) with respect to DNA twisting. Further discarding the initial 100 ns as equilibration, two blocks containing 200 ns from each sampling window were created, and WHAM method was applied to test for convergence. The procedure showed negligible deviation (< 5%) of the calculated PMF profiles, see Figure S6 for information on how the PMF profiles change with respect to the length of umbrella windows. The total simulation time was 11.5 μs for each system.

### Elastic force constants analysis

The PMF profiles are subjected to a quadratic regression analysis in MatLab to obtain the twisting force constants, K (kcal/mol deg^2^). The quadratic regression is performed for the entire profiles and for the regions corresponding to a ∆tw of ± 2°. Comparison of the quadratic fits indicates the presence of nonlinear effects in DNA response to torsional strain. In addition, quadratic regression is also applied for overtwisting and undertwisting individually to highlight the asymmetry of the PMF profiles.

The derived force constants are used to calculate the torsional modulus according to the isotropic rod model, given by Eq. (1); where T is the torque that results from a change in twist ∆ϴ over a specific length L (here L = 0.34 nm). From the torsional modulus one could derive the torsional persistence length P using Eq. (2), where k_B_T at room temperature corresponds to 4.224 pN nm.

1 $$T=K\Delta \theta =C\Delta \theta /L$$

2 $$P=C/{k}_{B}T$$

### Conformational analysis

The recorded trajectories are processed in CPPTRAJ^43^ program from AMBERTOOLS 16 software package. Subsequently, Curves+ and Canal programs^44^ are used to derive the helical parameters, backbone torsional angles and groove geometry parameters for each trajectory snapshot extracted at 1 ps intervals. This provides complete time-dependent information on the impact of the MafB-DNA complexation on DNA response to torsional stress.

### Contacts network analysis

Analysis of the protein-DNA contacts network is performed using CPPTRAJ^43^ program from AMBERTOOLS 16 software package, for each trajectory snapshot extracted at 1 ps intervals. We exclude the protein-DNA contacts that are present for less than 10% of the time in one umbrella window. We characterize the protein-DNA interactions by the pairs of residues, dividing the contacts into ‘specific’, formed between the protein side chains and DNA bases, and ‘non-specific’, formed with at least one of the molecules’ backbones. For each pair of protein-DNA residues we sum up all hydrogen bonds, salt bridges, and hydrophobic (apolar) interactions (Figure S15). The contribution of each type of contact is set to 1, for simplicity, since the energy cost of hydrogen bonds, salt bridges, and hydrophobic interactions varies greatly, depending on the nature of the atoms involved, the bond geometry and the surrounding environment. The limit of a direct interaction was set up to be ≤ 4 Å for a hydrogen bond between the relevant heavy atoms, and the angle limit was set up to ≥ 135° at the intervening hydrogen atom. For a salt bridge interaction, the limit was set up to 4.0 Å between the end-group nitrogen of lysine and arginine, and DNA phosphate group. A hydrophobic interaction was defined as a “dry” contact ≤ 6 Å between the centres of mass of hydrophobic residues (Ala, Ile, Leu, Met, Phe, Trp, and Cys) and DNA bases. The time series of MafB-DNA interactions allow construction of the dynamic contacts maps for specific and non-specific contacts, characterizing the stability and binding specificity of the MafB-DNA complex at various degrees of positive and negative torsional stress.

### Additional information

MatLab software was used for post-processing and plotting of all data. USCF Chimera^45^ was used for creating molecular graphics.

## Results and discussion

### Impact of MafB-MARE complexation on potential of mean force of DNA twisting

To address the impact of torsional stress on TF-DNA complexation we study two systems—the MafB-DNA complex (PDB ID: 4AUW)^20^ and free DNA in B-form. The DNA sequence in both systems, ‘GGTAAT**TGCTGACGTCAGCA**TTATGG’, contains the MARE motif in bold. The crystal structure of the MafB-DNA complex shows no major DNA deformation, which justifies the usage of B-form DNA as a reference state and, more importantly, allows us to decouple the impact of writhing and twisting on mechanisms of protein-DNA recognition and complex stability under supercoiling transitions. Following unrestrained molecular dynamics, we perform a cascade umbrella sampling, using the twist restraint, which controls the total twist of a DNA molecule. By applying the twist restraint to the 14 b.p. MARE-region, we gradually increase (overwinding) or decrease (underwinding) the total twist by 0.5°/b.p., starting from the relaxed state to a maximum of ± 5°/b.p, which corresponds to a change in supercoiling density of ± 0.15, to obtain the potential of mean force (PMF) of DNA twisting free energy as a function of average b.p. twist. We limit DNA twisting extremes to ± 5°/b.p. to avoid DNA melting, b.p. opening and flipping, which have been observed in computer simulations at higher degrees of torsional stress (~ 7°/b.p.)^46,47^. The b.p. of the restrained MARE-regions, in free and MafB-bound DNA, remain intact during both over- and undertwisting, although for some b.p. we observe occasional opening angles > |30°| and stretching >|0.8 Å| for less than 1% (Figure S3B) and 0.3% (Figure S4B) of the trajectories, respectively. We also observe no significant bending for free DNA (Figure S5A) with a smooth decrease in the bending angle when going from underwinding to overwinding. For MafB-bound DNA bending becomes more noticeable at higher degrees of both underwinding and overwinding (Figure S5B), reflecting the DNA groove geometry adjustments due to the protein presence (see section Impact of Torsional Stress on MafB-DNA Contacts Network). We would like to emphasize that the chosen torsional range shall be seen as an approximation of the extreme changes in local supercoiling that can arise, for example, in the proximity of transcription start sites, where DNA twisting plays a major role^5,48,49^. Moreover, the chosen torsional range allows us to computationally test hypotheses and collect mechanistic insights on: (1) the role that MafB, a specifically bound BZIP transcription factor, plays in the regulation of DNA supercoiling transitions; and (2) the role changing torsional restraints on DNA play in the regulation of DNA recognition by BZIP transcription factors. The actual range of local supercoiling changes in vivo remains to be discovered experimentally.

The observed B-like conformation of free and MafB-bound DNA under torsional stress, even at higher twisting regimes (> ± 4°/b.p.), appear at odds with the torque-induced behaviour of single long DNA molecules (kilo b.p. level) observed experimentally with magnetic and optical tweezers^50–52^. These experiments report the torque-induced elongation of DNA molecules, which is consistent with transition of B-DNA into alternative forms such as P-, L- or Z-DNA, depending on the sequence and torque regime at significantly smaller torsional stresses^53,54^. This seeming discrepancy between our computational results and experimental observations may arise from sequence specific effects and differences in DNA lengths. Long DNA molecules may contain torsionally-stiff and torsionally-flexible regions, as well as regions that melt easier (A/T-rich), and regions that are more stable (G/C-rich), which all will respond differently to torque. Moreover, in long systems, cooperative effects such as DNA denaturation, may increase the effect of DNA conformational conversion. Plausibility of the above effects is supported by the experimental evidence: a crystal structure of an extremely torsionally-stressed 2D triangular self-assembled DNA lattice (PDB ID: 5EOS)^33^, where one of the branches experiences about 4°/b.p underwinding while maintaining a B-like conformation.

**Figure 1:**
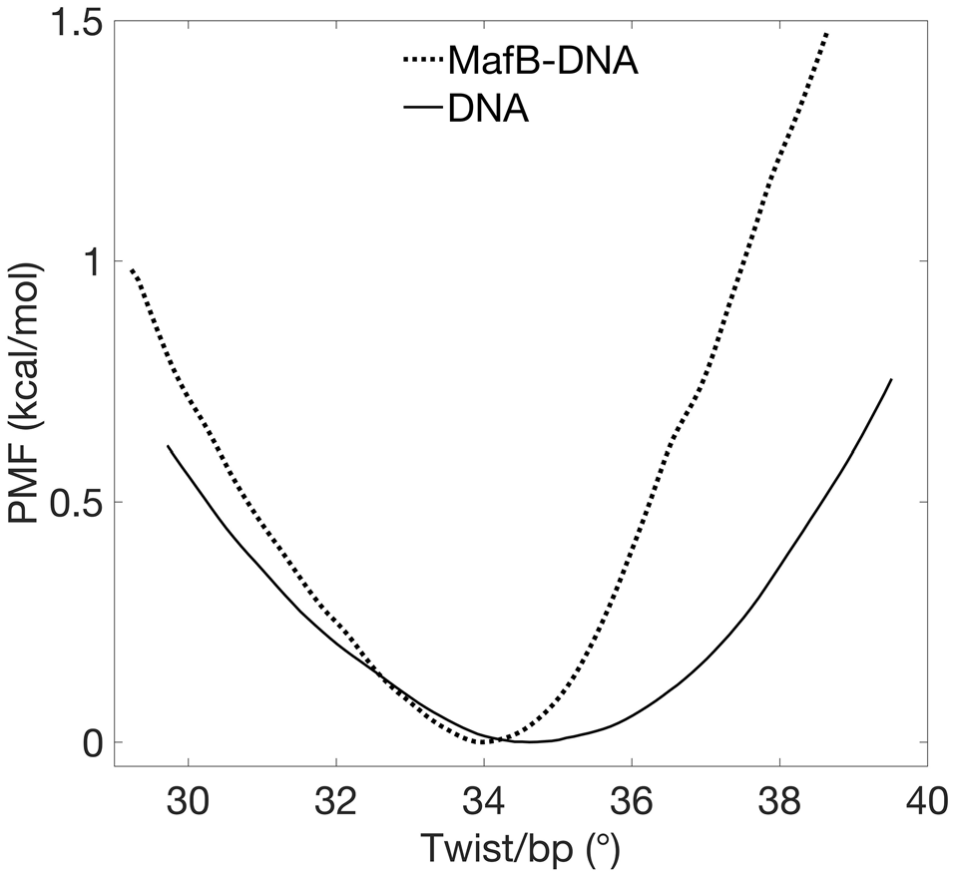
PMF profiles with respect to average twist per base pair step in free and complexed MARE-DNA.

The PMF profiles (Fig. 1) show that the energy cost for DNA twisting asymmetrically increases in the presence of MafB, where overtwisting becomes noticeably more unfavourable. In addition, MafB prefers binding to a slightly underwound MARE motif compared to free DNA (∆ twist = − 0.6° per b.p.; ~  − 8° for the 14 b.p. sequence; Table 1). For a more informative comparison, we use quadratic regression to calculate the torsional force constants for the PMF profiles. As the PMF curves are not symmetrical around the minima, we also derive the torsional force constants for overwinding and underwinding regimes individually (Table 1). The estimated force constants show that an additional 2.9 kcal/mol has to be paid to overwind complexed DNA by 5°/b.p. (65° in total) with respect to free DNA. In contrast, for the same degree of underwinding the energy difference is much smaller, 1.1 kcal/mol. The derived torsional force constants, of 0.06 and 0.11 kcal/mol × degrees^2^ for free and complexed MARE-DNA correspondingly, obtained from local quadratic regression (∆tw of ± 2°; Figure S7) of the PMF profiles can be used to calculate the torsional moduli and the persistence lengths (see Eqs. (1) and (2) in “Methods” section). The torsional moduli (Table 1) of 442 and 853 pN × nm^2^, for free and complexed MARE-DNA correspondingly indicate that DNA in complex with the transcription factor becomes nearly twice more rigid, which in turn suggests that MafB can modulate supercoiling transitions.

**Table 1:** Calculated average relaxed twists, torsional constants ‘K’ (overall), ‘K−’ (undertwisting regime), ‘K+’ (overtwisting regime), torsional moduli ‘C’, and torsional persistence lengths ‘P’ for MARE-DNA alone and in complex with MafB.

	DNA	MafB-DNA
Relaxed tw (°)	34.6	34.0
K (kcal/mol deg^2^)	0.057	0.11
K + (kcal/mol deg^2^)	0.069	0.11
K− (kcal/mol deg^2^)	0.039	0.055
C (pN nm^2^)	442	853
P (nm)	107	207

We want to emphasize that the derived numbers characterising the torsional rigidity of free and MafB-bound MARE-DNA are model and force field dependent, thought the calculated torsional modulus of free DNA is in the range of experimental measurements using single molecule techniques, which yielded the torsional modulus of 410 ± 30 pN × nm^2^^50,52^. Moreover, the observed doubled torsional rigidity of MafB-bound MARE-DNA, we believe, is a local effect created by the protein, however highlighting an important trend that a specifically bound protein can inhibit the propagation of supercoiling thus likely leading to the accumulation of supercoiling at the flanking sites of MARE rather than within the TF-bound region, which can contribute to the regulation of transcription of near-located genes.

### Sequence-specific response of individual base pair steps to torsional stress

**Figure 2:**
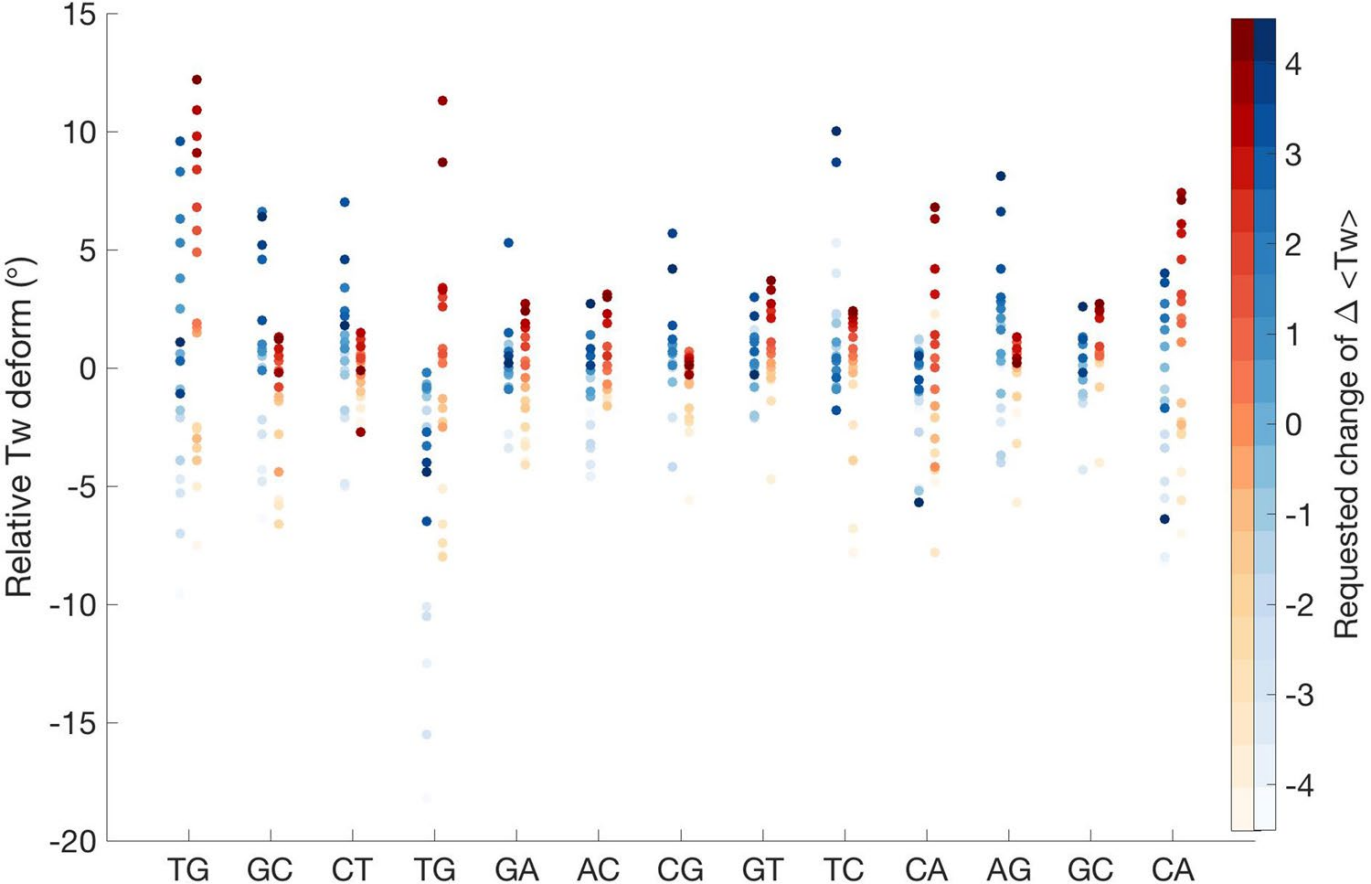
Changes of twist angles for the restrained MARE-region in free and complexed DNA as a function of the requested average change of twist per base pair step, indicated by a colourbar to the right.

To explain the mechanism of the induced rigidity by MafB complexation, we analyse the response of the individual b.p. steps to torsional stress (Fig. 2). In accordance with our previous studies^9,10^, the pyrimidine-purine steps, TpG and CpA steps, of C-TpG-A and T-CpA-G motifs exhibit major torsional flexibility in free DNA, effectively absorbing both negative and positive torsional stress. The two tetranucleotide motifs are symmetrical, belonging to each half-site of the MARE motif (TGCTGACGTCAGCA). However, In the presence of MafB, the sequence-specific response changes, the TpG and CpA steps become torsionally rigid. Twist distributions (Figure S9) for the steps, which show twist bimodality in free relaxed DNA, exhibit a high twist state in the protein environment. During DNA overwinding, these steps remain passive, as they are unable to increase their twist further. The contribution of the TpG and CpA steps to efficient DNA underwinding is also limited. Instead, other b.p. steps that are less flexible in free DNA are forced to modify their twist, resulting in the increased energy cost for DNA twisting. We exclude the first and the last b.p. step of the restrained region (TGCTGACGTCAGCA) from the analysis since the variation of their twist values may result also from the boundary effects. For the comparison of twist distributions for the restrained MARE-region for the relaxed, overwound (+ 4.5°), and underwound (− 4.5°) states see Figure S9.

We consider further the protein role in the modified DNA sequence-specific response to torsional stress by analysing other rotational and translational helical parameters (Figures S10–S14). Consistent with the previous publications^9,10^, torsional stress also induces changes in roll, shift, slide and to some extend rise, whereas tilt remains unaffected. For free DNA, we observe that roll (Figure S10) decreases uniformly as twist increases. For MafB-bound DNA, the response is similar, however the roll distributions are narrower, indicating a more rigid system. Together with twist, the greatest impact of torsional stress and MafB binding is observed for shift and slide (Figures S12–13). Upon association with the MARE-region, MafB induces local structural adjustments of the double helix. In particular, the TpG step (TGC**TG**AC), which hydrophobically interacts with Tyr251, Ala252, and Cys255 of RxxNxx**YA**xx**C**R-motif, exhibits a more positive slide and negative shift—the G-C b.p. is effectively pushed towards the DNA minor groove (Figure S12–13). The same behavior is observed for the symmetric CpA step (GT**CA**GCA) in the other MARE-half site. This allows the protein to have tighter hydrophobic contacts with the T-A b.p. of the MARE-half sites. Since the helical parameters shift, slide and twist are structurally coupled via the BI/BII backbone conformational transitions^9,10,13^, the protein-induced negative shift and positive slide locks the TpG and CpA dinucleotides in a high twist substate. In addition, during undertwisting, for MafB-bound DNA the first four b.p. steps of the first MARE half-site (**TGCTG**AC) exhibit a decrease in slide, which leads to a loss of contacts between monomer 2 and DNA as described in the next section.

### Impact of torsional stress on MafB-DNA contacts network

**Figure 3:**
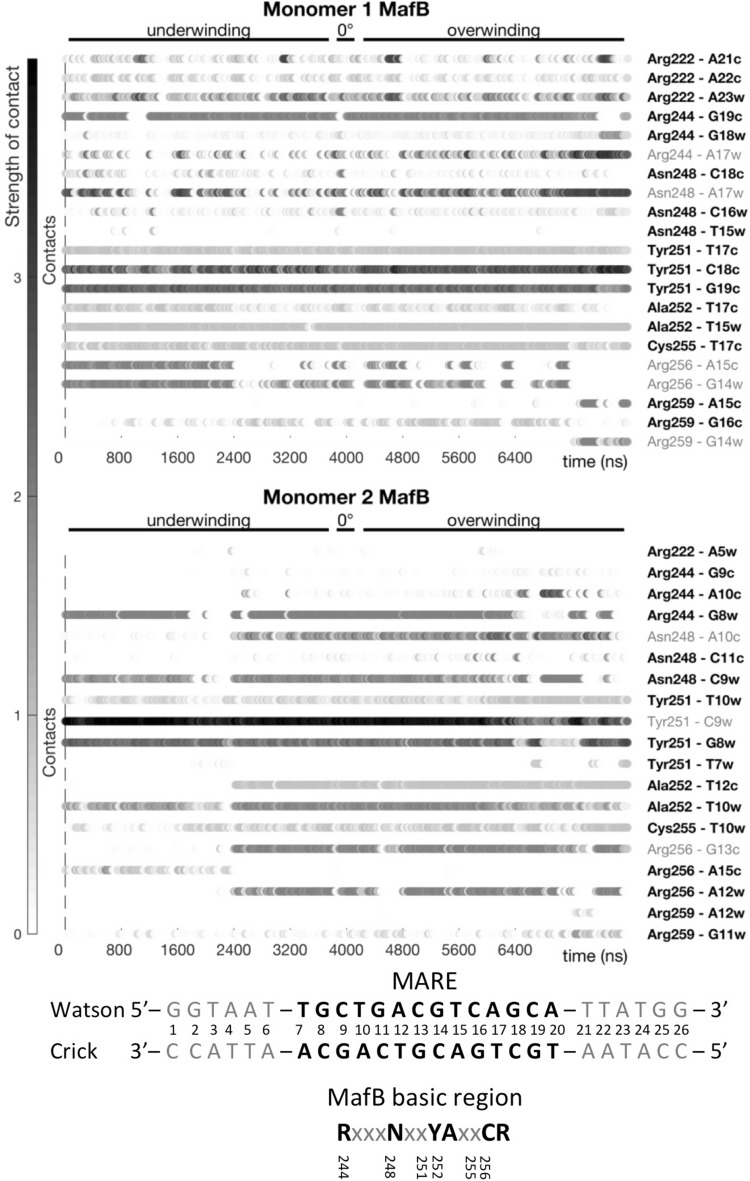
Dynamic interactions maps of specific MafB-DNA contacts at different degrees of torsional stress. The contacts between pairs of residues are characterized by strength and occurrence. Torsional stress denoted as ‘underwinding’ represents changes from − 5°/b.p. to − 0.5°/b.p.; and ‘overwinding’—from 0.5°/b.p. to 5.0°/b.p. Contacts in bold change insignificantly (change in contact strength < 1) with changing torsional stress.

We monitor the evolution of the MafB-DNA contacts network to estimate the structural impact of changing torsional stress on the protein-DNA complex. Since the MafB-MARE recognition process follows the direct readout mechanism, we hypothesize that the number of contacts represents the complex stability. We characterize the protein-DNA interactions by pairs of residues, dividing the contacts into ‘specific’, formed between the protein side chains and DNA bases, and ‘non-specific’, formed with at least one of the molecules’ backbones. For each pair of protein-DNA residues, we sum up all hydrogen bonds, salt bridges, and hydrophobic (apolar) interactions. The contribution of a single bond of each type is set to 1 (Figure S15), for simplicity, since the energy cost of every type of bond vary greatly depending on the nature of atoms involved, the bond geometry, and the surrounding environment. The time series of MafB-DNA interactions allow the construction of dynamic contacts maps for specific (Fig. 3) and non-specific contacts (Figure S16), characterizing the stability and the binding specificity of the MafB-DNA complex at different degrees of positive and negative torsional stress.

**Figure 4:**
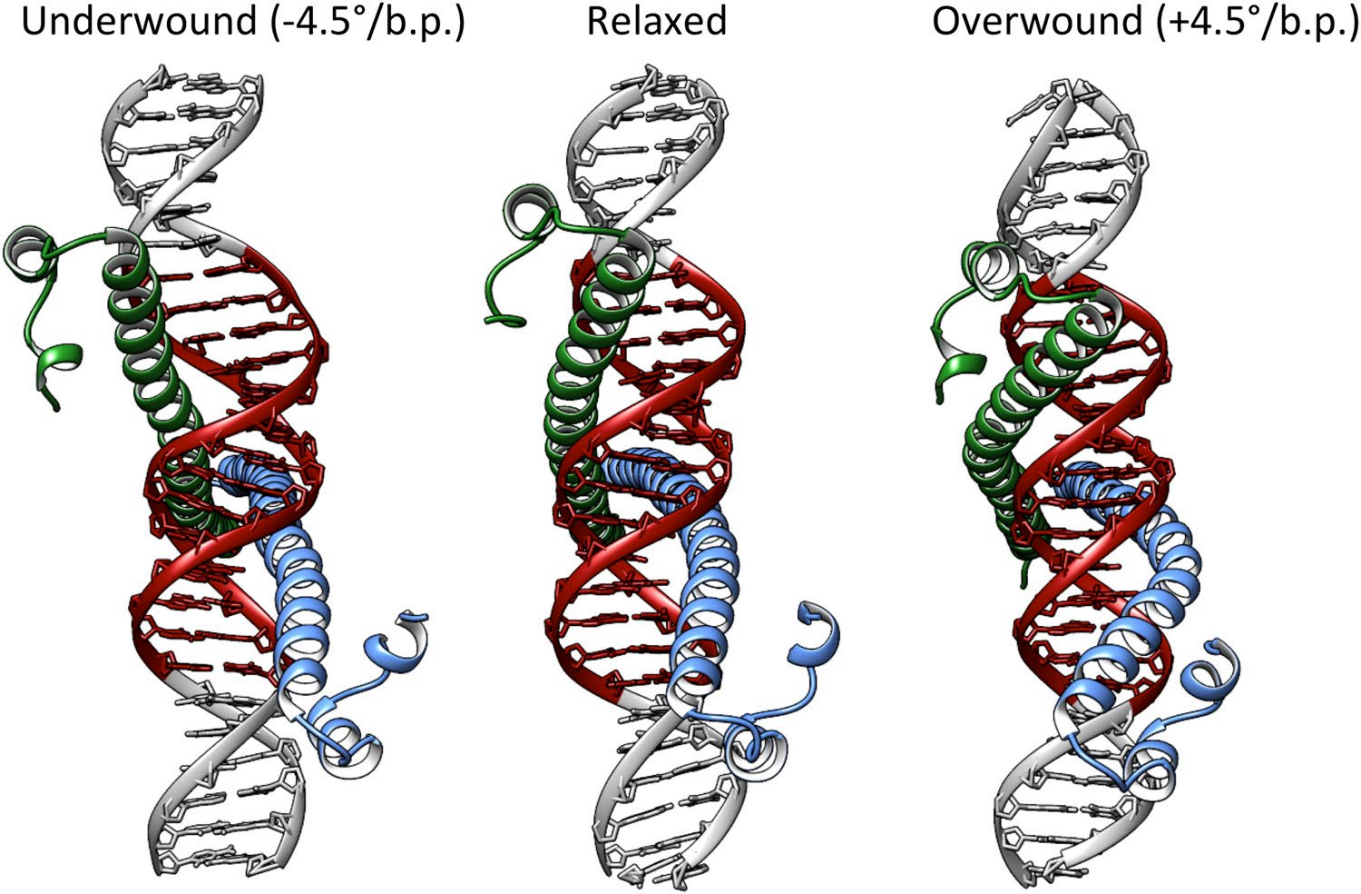
Structural changes in the BZIP domain of the MafB-dimer at the underwound and overwound states, with respect to the torsionally relaxed state.

The dynamic contacts maps show that despite torsional stress, MafB maintains the majority of the specific contacts with the MARE-binding site. However, we observe some changes in the intermolecular contacts network during DNA underwinding. The MARE-half site on Watson-strand absorbs most of the negative torsional stress, making the T12c and G13c bases inaccessible for MafB monomer 2. This leads to the loss of specific contacts: Ala252(2)-T12c and Arg256(2)-G13c, at higher levels of underwinding < − 2.5°/b.p. Instead, a compensating contact Arg256(2)-A15c is formed. The MARE-half site on Crick strand exhibits a tighter interaction with MafB monomer 1, which effectively makes it more torsionally rigid. Furthermore, DNA underwinding stabilizes two specific contacts, Arg256(1)-G14w and Arg256(1)-A15c. The most noticeable change during extreme DNA overwinding, > 4°/b.p., is formation of a specific contact, Arg259(1)-G14w. The dynamic contacts maps for the non-specific contacts (Figure S16) mirror the trends of the specific contacts, namely, the mentioned arginine residues gain or lose, respectively, contacts with the DNA backbone. We also observe that to maintain the contacts with torsionally stressed DNA, MafB modifies the structure of its DNA binding domains (Fig. 4). At the higher degree of underwinding, <  − 4.0°/b.p., as DNA major groove becomes wider and deeper  (Figure S17), the long alpha-helices bend away from DNA. For the higher degree of overwinding, > 4.0°/b.p., with DNA major groove becoming relatively shallow and narrow (Figure S17), the helices buckle away from the major groove (For more details see Supplementary movies). Thus, although specific binding of MafB, and BZIP proteins in general, to relaxed DNA induces no major DNA deformations, our computations show that the transcription factors can exploit local sequence-specific deformations and still maintain sequence-specific contacts with the response element by undergoing conformational changes. We also observe a cooperative communication mechanism between the protein monomers, where one monomer compensates for the loss of contacts between another monomer and torsionally stressed DNA, which we propose, is characteristic for BZIPs due to their flexible DNA binding domains.

## Conclusions

Torsional stress constitutes a major regulatory mechanism of eukaryotic transcriptional control. While propagating along the chromatic fibre, torsional stress introduces local conformational changes of the DNA double helix that modulate the organization of nucleosomes and the opening of promoters. Torsional stress propagates by locally, a few hundred of b.p., twisting DNA, and globally by creating loops and knots. The molecular mechanism of how torsional stress affects the selective binding of transcription factors to DNA remains elusive. In this study, we have used microsecond long umbrella sampling simulations coupled to a torsional restrain that controls the total twist of a DNA molecule, to provide first molecular mechanistic insights into the impact of torsional stress on transcription factor-DNA complexation, employing human BZIP factor, MafB-DNA complex. Starting from a fully relaxed state, we have gradually underwound and overwound DNA in the complex with MafB and in free B-DNA conformation, used as a reference state, by a maximum of ± 5° per b.p. step. We have calculated the potential of mean force and analysed the individual contribution of b.p. steps to the absorption of torsional stress, as well as the evolution of specific and non-specific contacts as DNA experiences negative and positive torsional stress.

Our results show that the presence of specifically bound MafB modifies the DNA sequence-specific response to torsional stress. The torsionally flexible dinucleotide steps in free DNA become torsionally rigid in the protein environment, as they are involved in specific protein-DNA contacts. Instead, other b.p. steps that are expectedly less flexible are forced to respond to changing torsional stress. MafB, to maintain the stable and selective binding to its DNA target, undergoes substantial conformational changes, where the BZIP long alpha-helical coiled-coil DNA binding domains adapt to the torsionally modified geometry of the double helix. This overall results in an asymmetric increase of free energy of DNA twisting transitions, relative to free DNA, where overtwisting becomes more energetically unfavourable.

However, we believe that torsional stress may affect differently the protein-DNA complexes with non-specifically bound BZIPs. In such complexes (e.g. PDB ID: 1A02)^55^, the factors interact mostly with the DNA backbone via salt-bridges, which modulates the geometry of DNA grooves making it more shape-complementary for the BZIP factors, but affect to a lesser extent shift and consequently twist values within the DNA binding partner. Our observations suggest an interesting and potentially important trend that BZIPs that specifically interact with DNA act as topological insulators, hindering the propagation of torsional stress along the chromatin fibre, thus leading to accumulation of DNA supercoiling at the flanking sites rather than within DNA–protein response elements. The stable specific binding of BZIP factors, we hypothesize, may initiate a formation of enhanceosomes, acting as pioneer factors that inhibit chromatin compaction, and contribute to the regulation of transcription of nearby genes.

## Supplementary information

Supplementary Information 1.

Supplementary Video S1.

Supplementary Video S2.

## Data Availability

All data generated and analysed in this study are available from the corresponding author upon request.
