# Supplementary material for: Specifically bound BZIP transcription factors modulate DNA supercoiling transitions
Source: Sci Rep. 2020 Nov 2;10:18795. doi: 10.1038/s41598-020-75711-4 (PMC7606469; doi:10.1038/s41598-020-75711-4)
Supplement: Supplementary file 1 — Supplementary Information 1. [file 41598_2020_75711_MOESM1_ESM.pdf]

SUPPORTING INFORMATION

Specifically Bound BZip Transcription Factors Modulate DNA Supercoiling Transitions

Johanna Hörberg and Anna Reymer*

Department of Chemistry and Molecular Biology, University of Gothenburg, Gothenburg 40530, Sweden

* To whom correspondence should be addressed. Email: anna.reymer@gu.se

Supplementary Figures

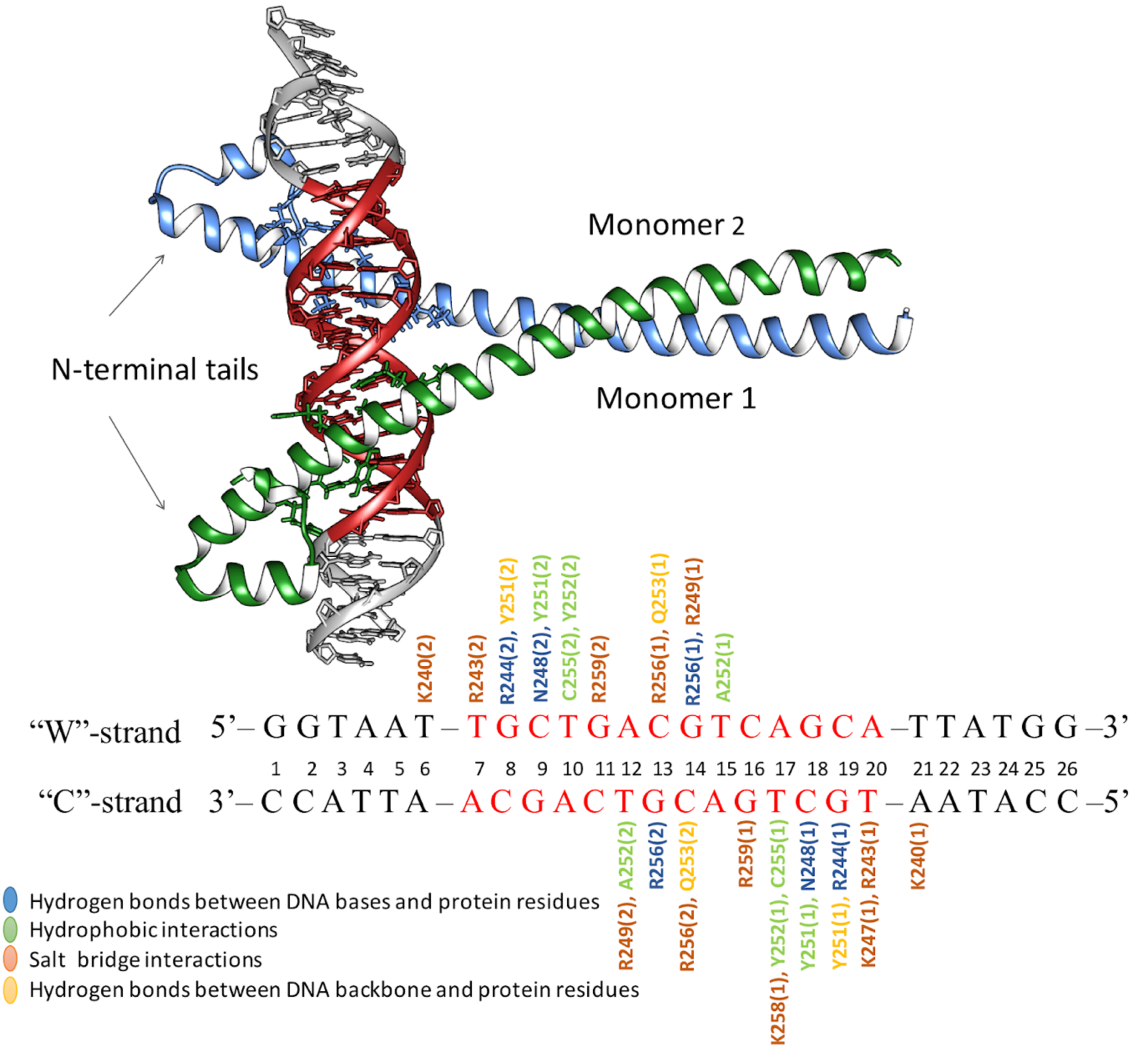

Figure S1. Crystal structure of MafB-DNA complex (PDB ID: 4AUW), containing the MARE region in red. The contacts between MafB and DNA that are present in the complex are highlighted.

MafB-DNA Contacts Involving the Conserved Six Residue Motif

Rxxx**N**xx**YA**xx**CR**

Specific
Hydrophobic
Nonspecific

**Prot. Res.**	**Monomer 1**	**Monomer 2**
Arg244	**G19c**	**G8w**
Asn248	**C18c**	**C9w**
Tyr251*	G19c<br>C18c<br>T17c	G8w<br>C9w<br>T10w
Ala252	T15w	T12c
Cys255	T17c	T10w
Arg256	C13w<br>**G14w**	**G13w**<br>C14w

*: Tyr251 stabilises the orientation of Arg244 and Asn248.

"W"-strand 5'– G G T A A T – T G C T G A C G T C A G C A –T T A T G G –3'
1 2 3 4 5 6 7 8 9 10 11 12 13 14 15 16 17 18 19 20 21 22 23 24 25 26
"C"-strand 3'– C C A T T A – A C G A C T G C A G T C G T – A A T A C C –5'

Figure S2. Intermolecular interactions between the DNA-recognizing motifs of MafB monomers and DNA, present in the crystal structure (PDB ID: 4AUW).

A

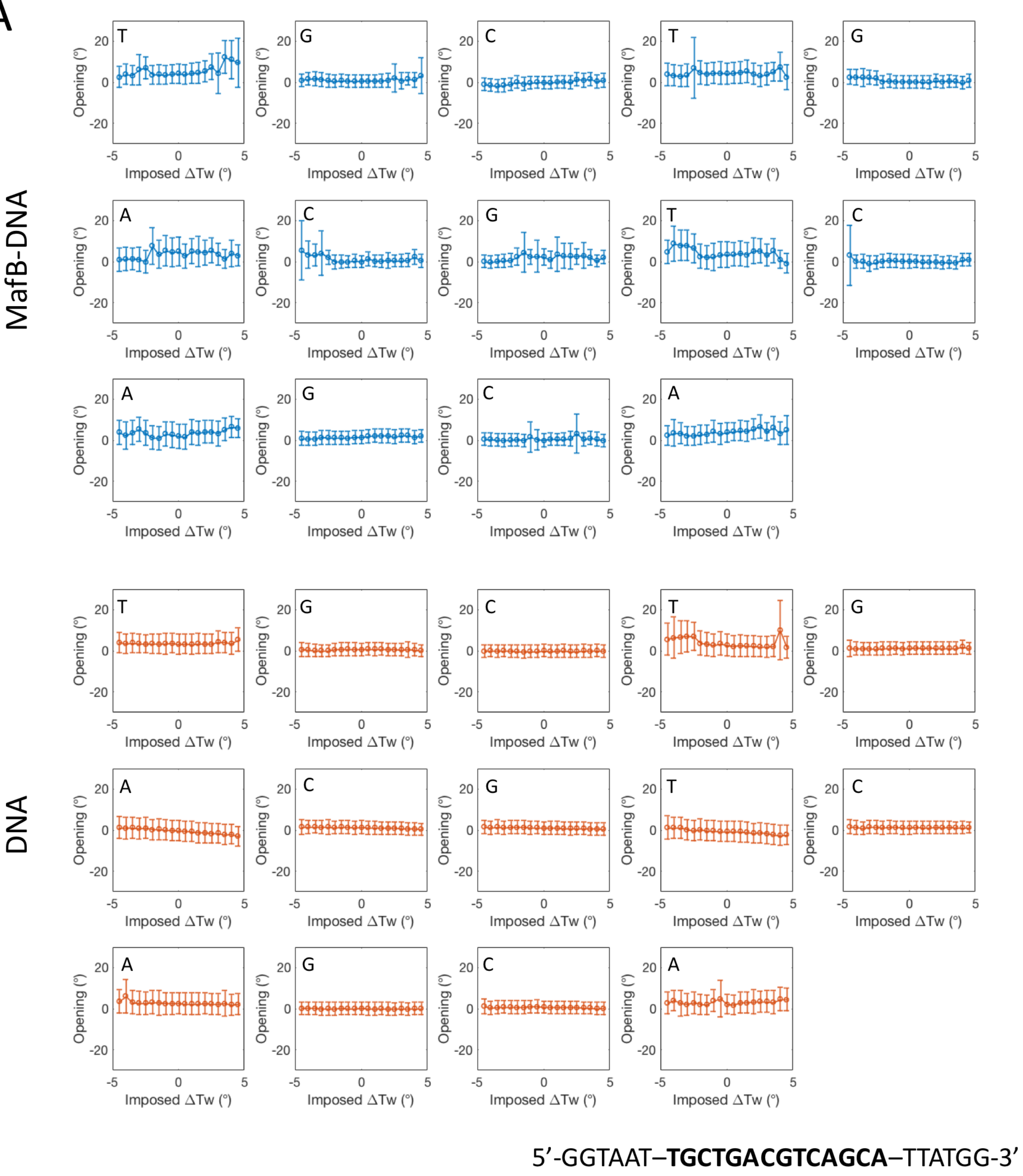
MafB-DNA
DNA
T
G
C
T
G
A
C
G
T
C
A
G
C
A
Opening (°)
Imposed ΔTw (°)
20
0
-20
-5
0
5
5'-GGTAAT–**TGCTGACGTCAGCA**–TTATGG-3'

B

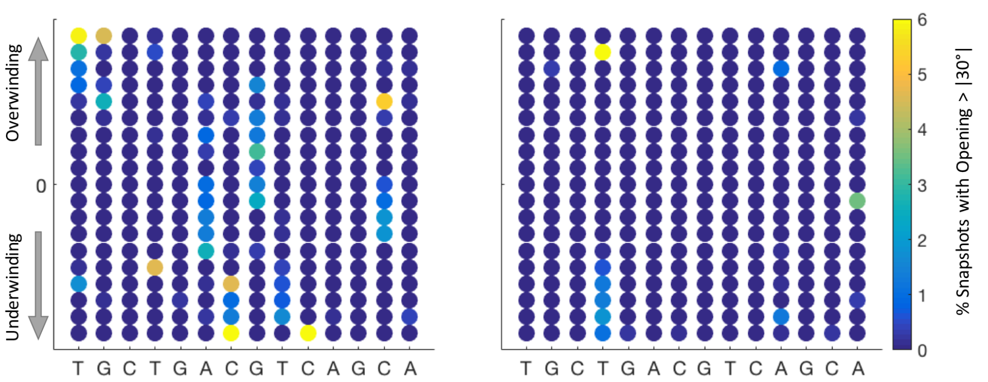
MafB-DNA
DNA
Overwinding
Underwinding
0
T G C T G A C G T C A G C A
T G C T G A C G T C A G C A
% Snapshots with Opening > |30°|
6
5
4
3
2
1
0

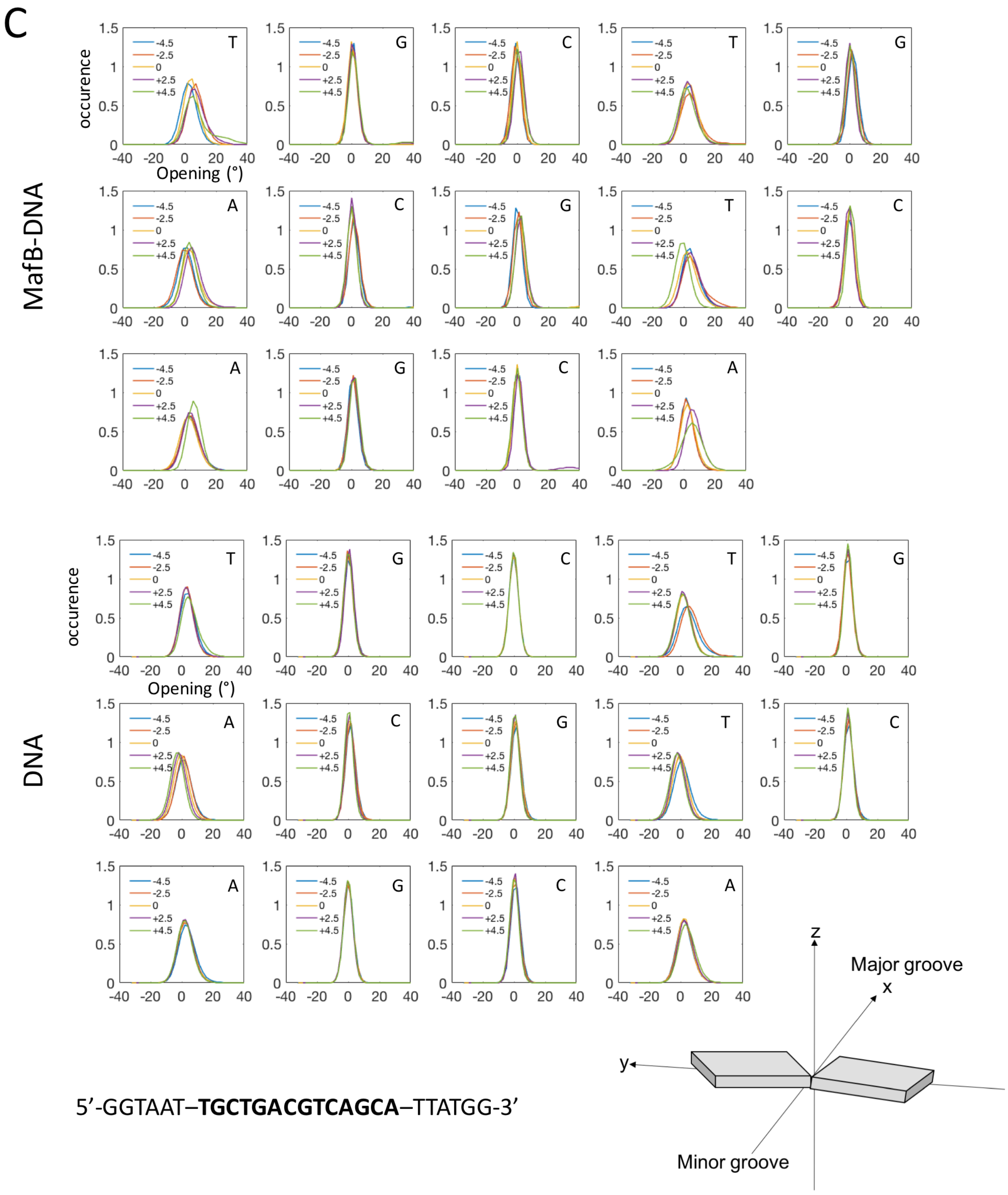

Figure S3. **A.** Average opening angle (°) and standard deviation of each base pair (b.p.) in the MARE sequence as a function of imposed twist for MafB-DNA (blue) and free DNA (orange). **B.** Colourmaps showing percentage of trajectory snapshots, where a b.p. has an opening angle > |30°|. **C.** Opening angle distributions for different torsional regimes (°/b.p. step).

A

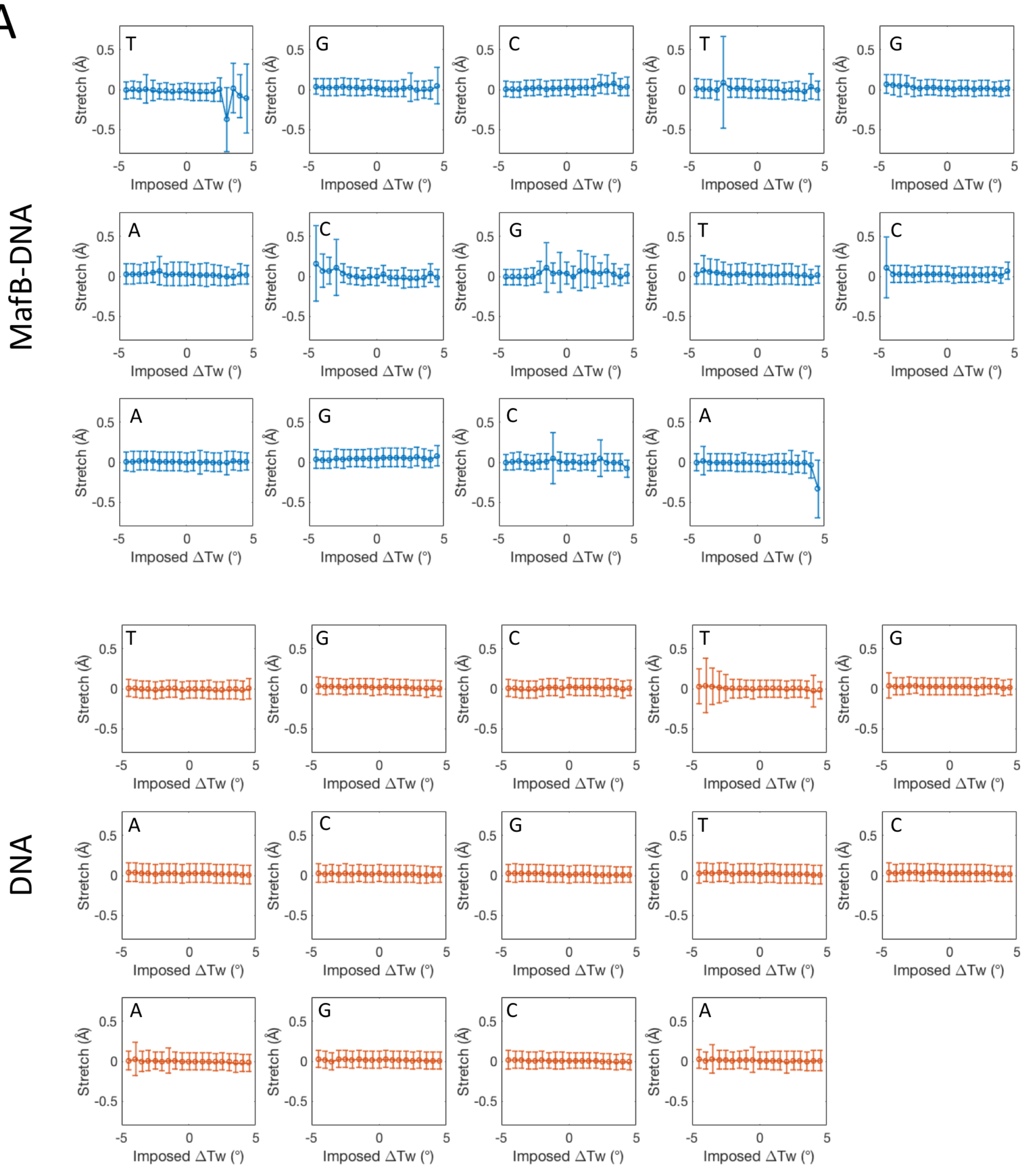

5’-GGTAAT–**TGCTGACGTCAGCA**–TTATGG-3’

B

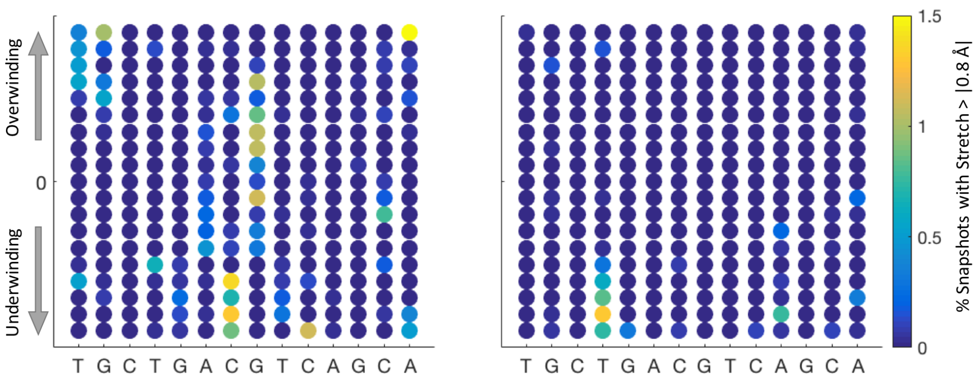

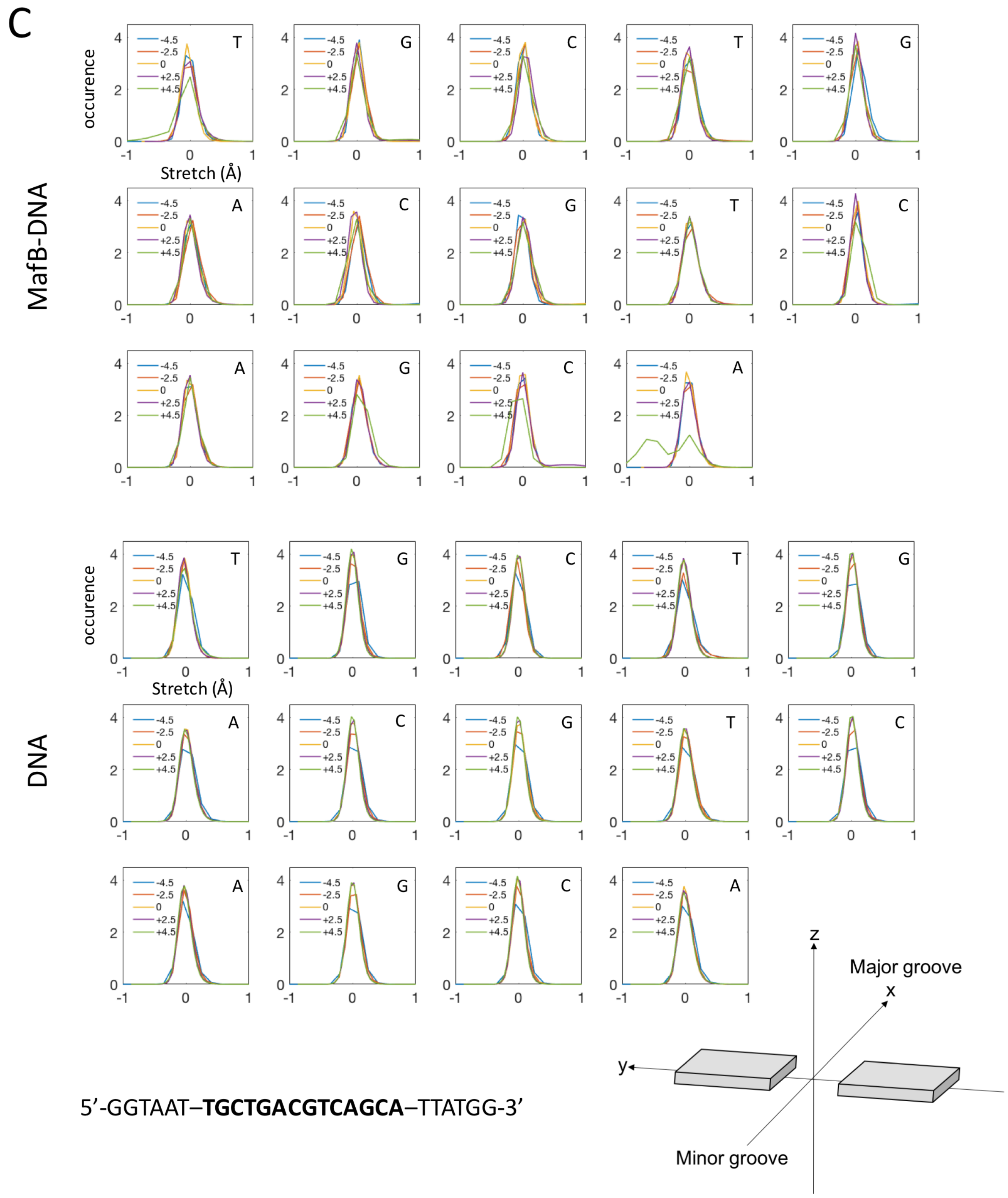

Figure S4. **A.** Average stretching (Å) and standard deviation of each base pair (b.p.) in the MARE sequence as a function of imposed twist for MafB-DNA (blue) and free DNA (orange). **B.** Colourmaps showing percentage of trajectory snapshots, where a b.p. exhibits a stretching distance > |0.8 Å|. **C.** Stretch distributions for different torsional regimes (°/b.p. step).

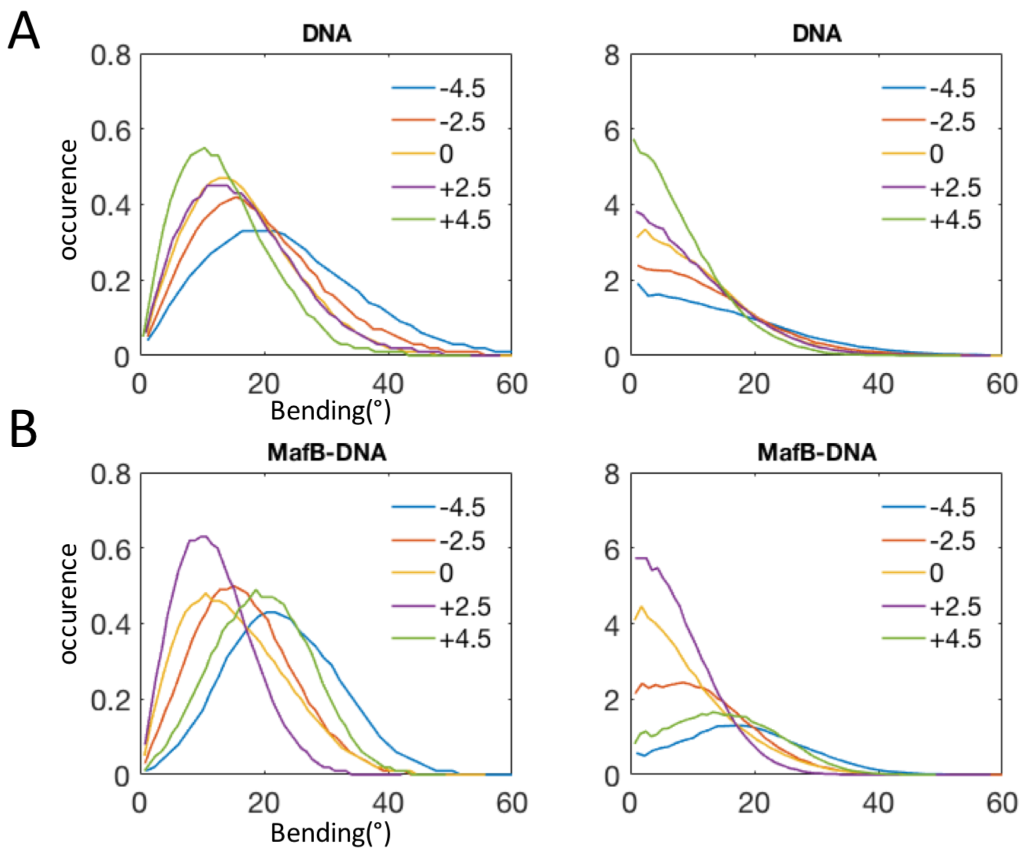

Figure S5. Bending distributions of the restrained MARE region for Mafb-DNA (upper panels) and free DNA (bottom panels). For the right-hand upper and bottom panels, the histograms have been divided by sin(***θ***), where ***θ*** corresponds to the bending angle at the middle of each histogram bin, to compensate for the increasing area of the spherical ring segment sampled as the bending angle increases.

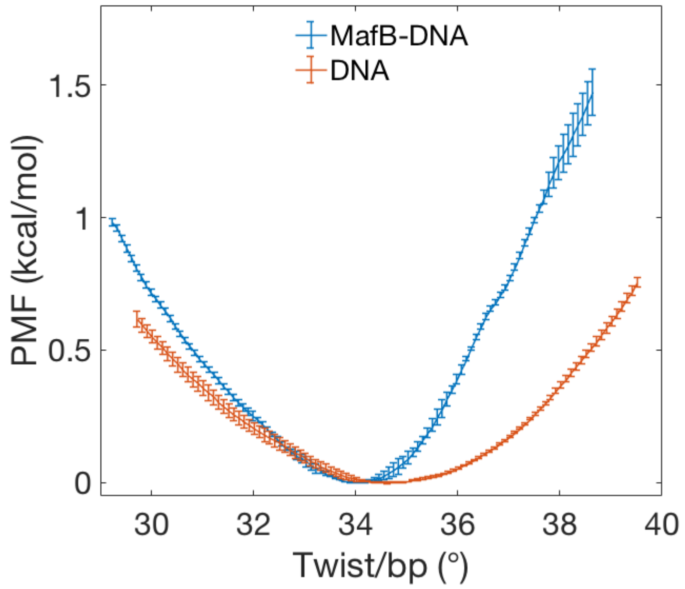

Figure S6. PMF profiles of DNA twisting transitions for MafB-DNA (blue) and free DNA (orange) with respect to average twist per base pair step. The standard deviation of going from 100 ns simulations to 500 ns simulations is illustrated as error bars.

A

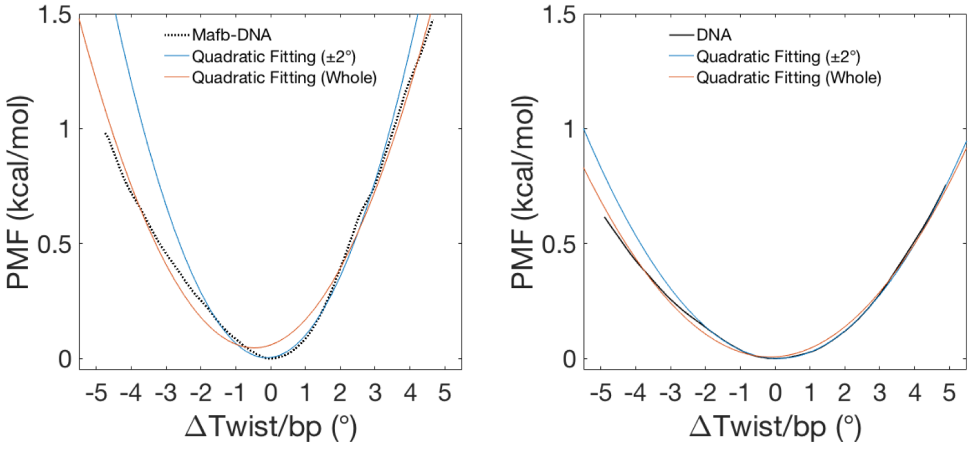

B

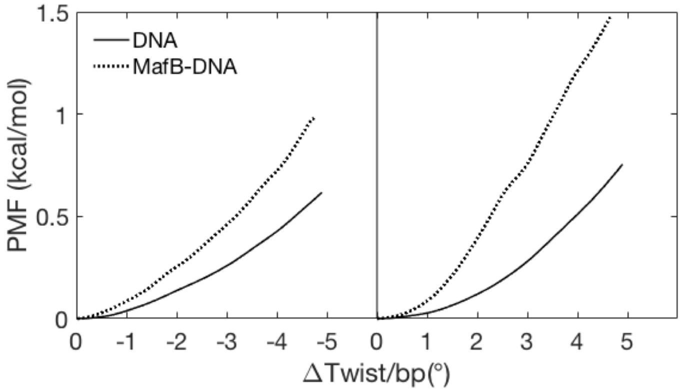

C

	DNA	MafB-DNA
Relaxed tw (°)	34.6	34.0
K (kcal/mol*deg^2)	0.057	0.11
K+	0.069	0.11
K-	0.039	0.055
C (pN nm^2)	442	853
P (nm)	107	207

***Figure S7.* A).** PMF profiles for MafB-DNA (left panel, dotted lines) and free DNA (right panel, black lines) as a function of ΔTwist/b.p. Quadratic regression is shown in blue lines (local fitting, ±2°) and orange lines (Global fitting). **B).** PMF profiles for underwinding (left panel) and overwinding (right panel) of free DNA (black lines) and MafB-DNA (dotted lines) illustrating the asymmetry of the profiles. **C)** Calculated average relaxed twists, torsional constants ‘K’ (overall), ‘K-’ (undertwisting regime), ‘K+’ (overtwisting regime), torsional moduli ‘C’, and torsional persistence lengths ‘P’ for MARE-DNA alone and in complex with MafB transcription factor.

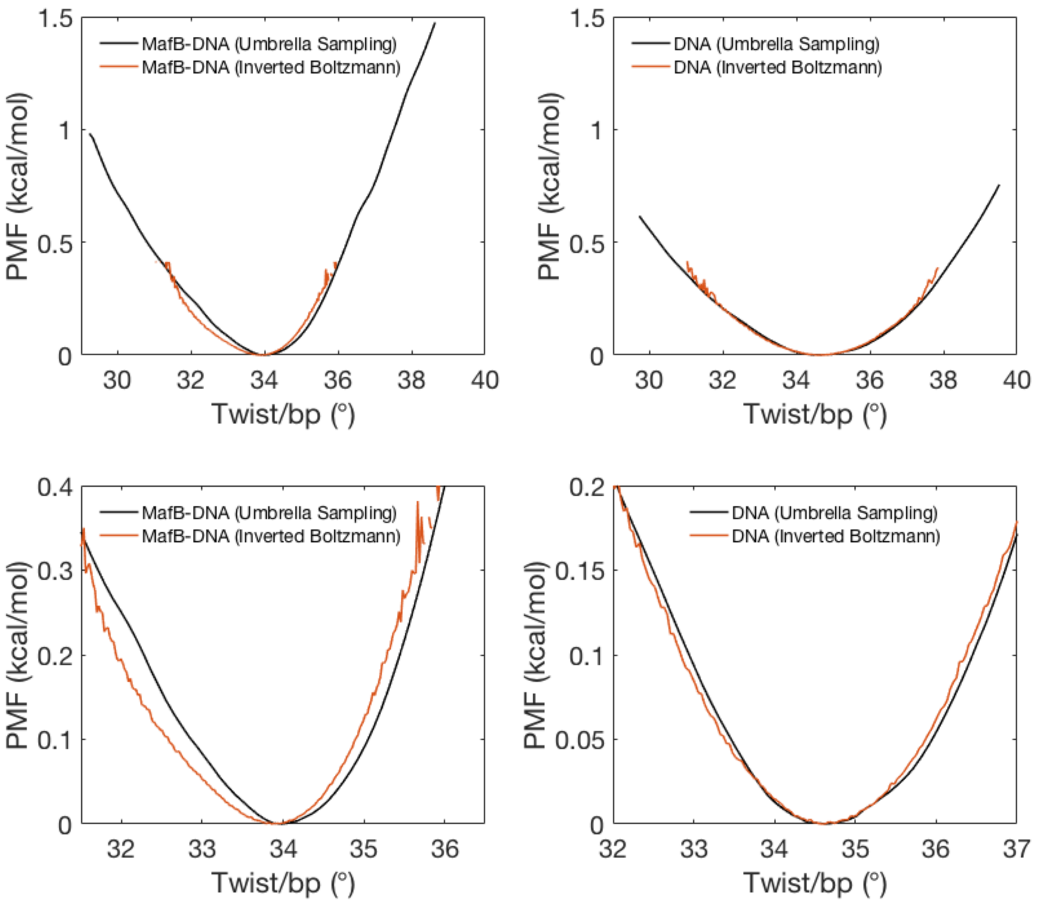

Figure S8. Comparison of PMF profiles derived from Umbrella Sampling (black) and Inverted Boltzmann (orange). PMF profiles for MafB-DNA are shown in the upper (±5°/b.p.) and bottom (zoom ±2.5°/b.p.) left-hand panels. PMF profiles for Free DNA are shown in the upper (±5°/b.p.) and bottom (zoom ±2.5°/b.p.) right-hand panels. The inverted Boltzmann profiles have been calculated using the equation –RT*ln(N), where RT is the product of the gas constant and temperature with a value of 0.593 kcal/mol at 25°C, and N is the distribution probabilities of the of the total twist of the restrained MARE region derived from unrestrained molecular dynamics trajectories of 500 ns.

The analysis is performed to validate the usage of a computationally more expensive umbrella sampling method for obtaining the PMF profiles of DNA twisting transitions. By comparing the PMF profiles, we conclude that the inverted Boltzmann method is able to overall recover the umbrella sampling derived PMF profile for free DNA, in particular in the range of small over- and underwinding deformations of ±2°/b.p. However, for MafB-bound DNA, the deviation between the inverted Boltzmann and umbrella sampling PMF profiles is larger, reflecting the differences in the conformational sampling in the unrestrained versus torsionally restrained DNA simulations.

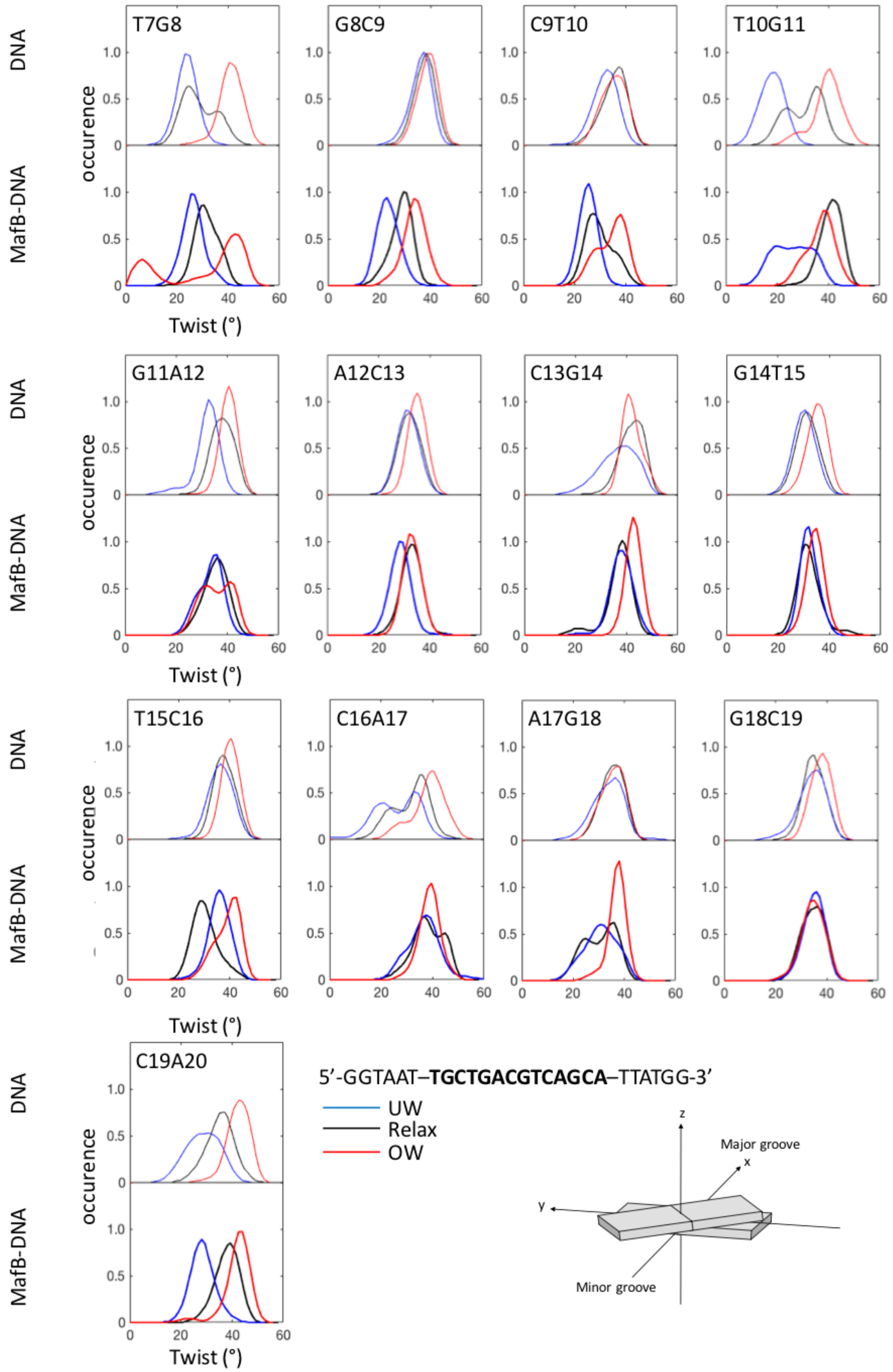

Figure S9. Twist distributions of the restrained MARE region for underwound (-4.5), relaxed and overwound (+4.5) state. MafB-DNA is denoted with thicker lines.

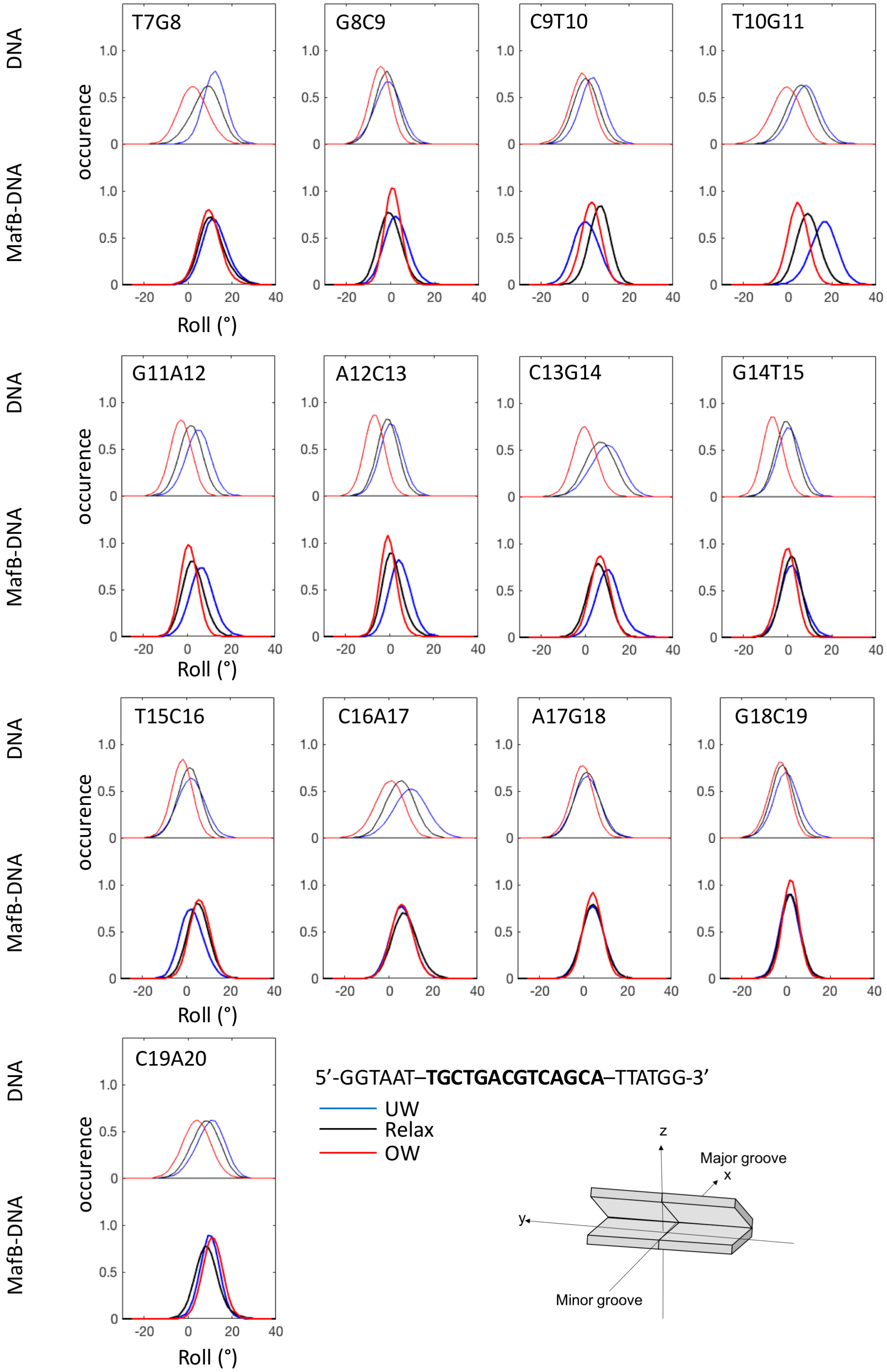

Figure S10. Roll distributions of the restrained MARE region for underwound (-4.5), relaxed and overwound (+4.5) state. MafB-DNA is denoted with thicker lines.

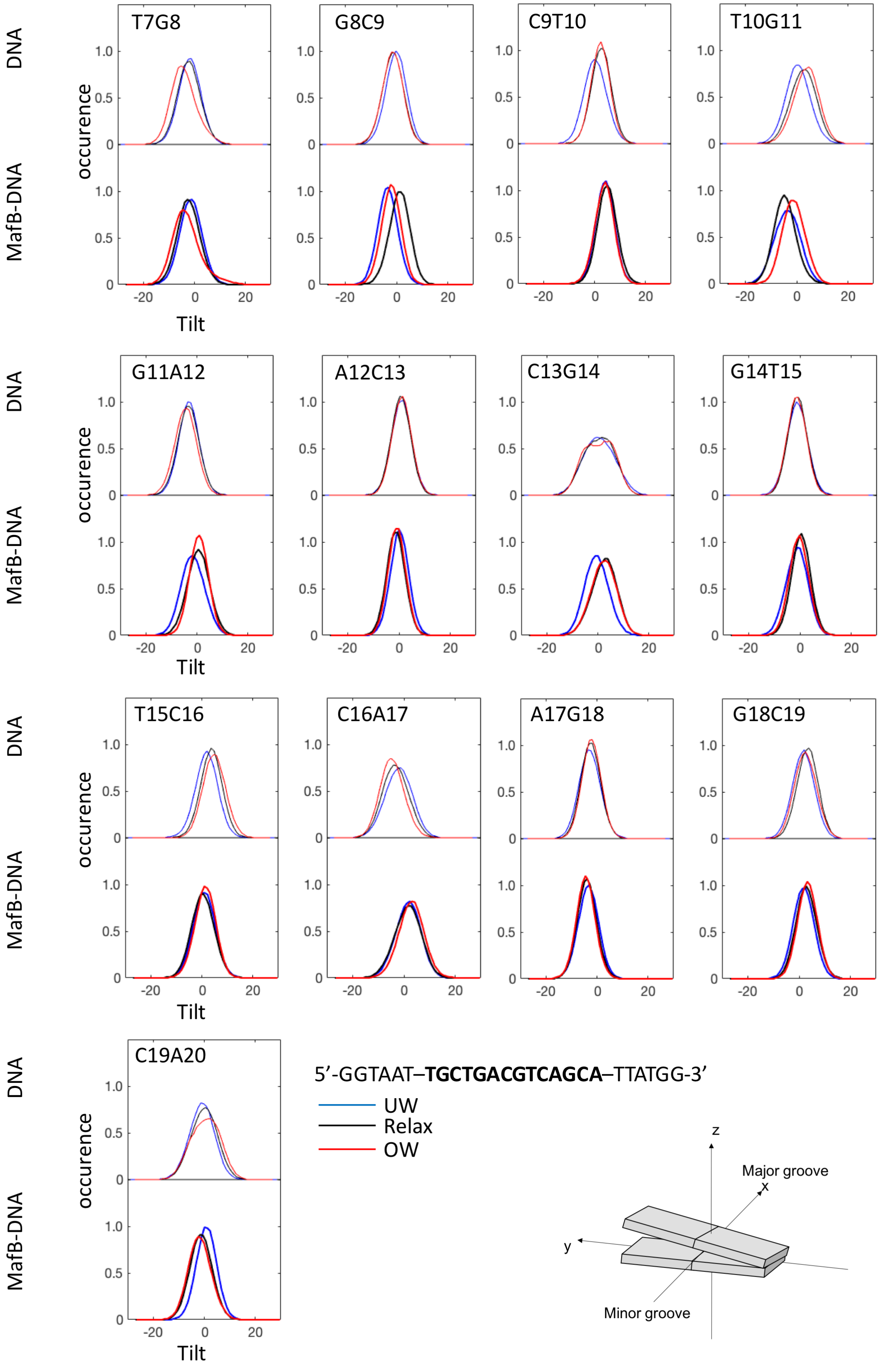

Figure S11. Tilt distributions of the restrained MARE region for underwound (-4.5), relaxed and overwound (+4.5) state. MafB-DNA is denoted with thicker lines.

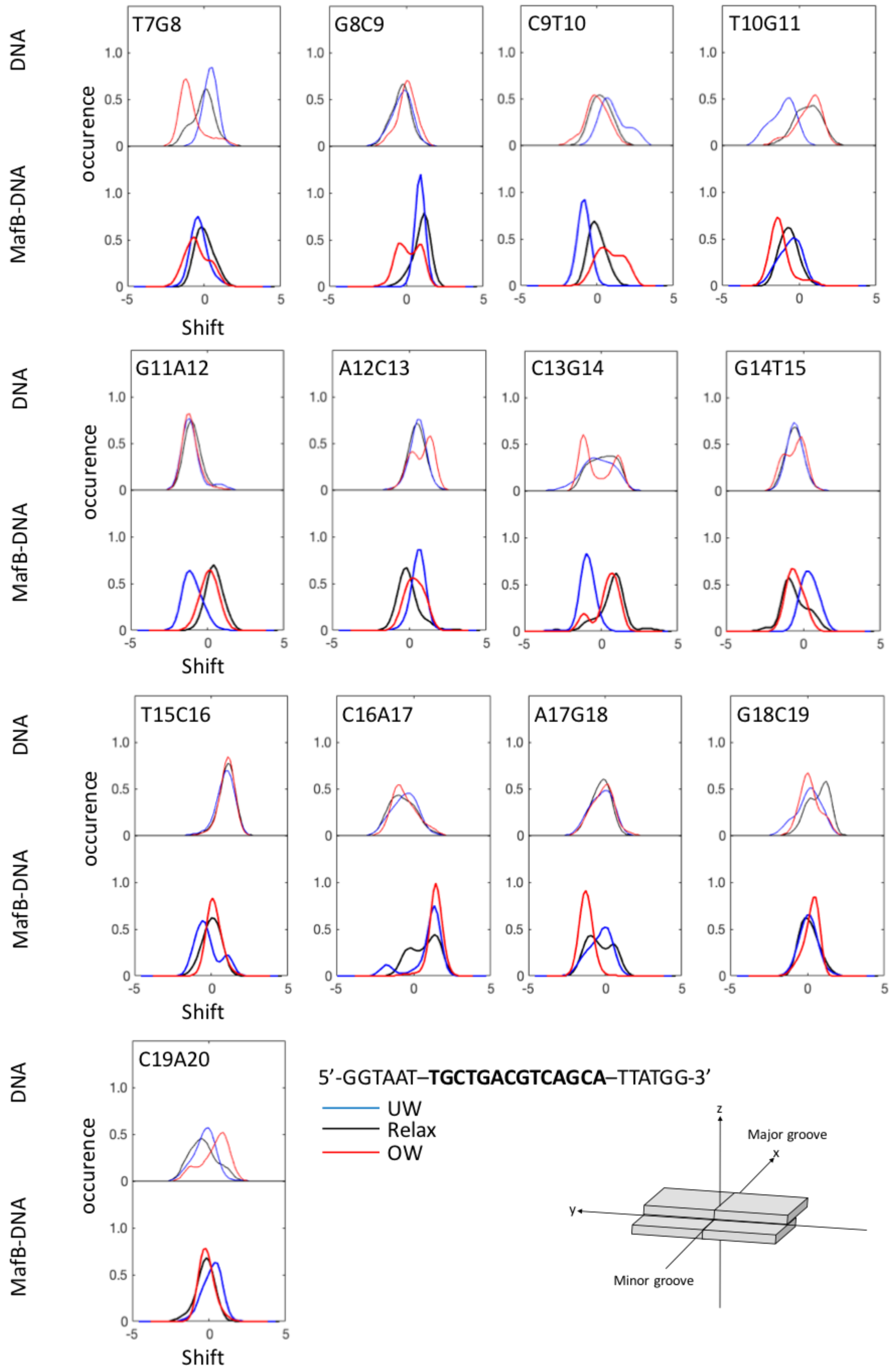

Figure S12. Shift distributions of the restrained MARE region for underwound (-4.5), relaxed and overwound (+4.5) state. MafB-DNA is denoted with thicker lines.

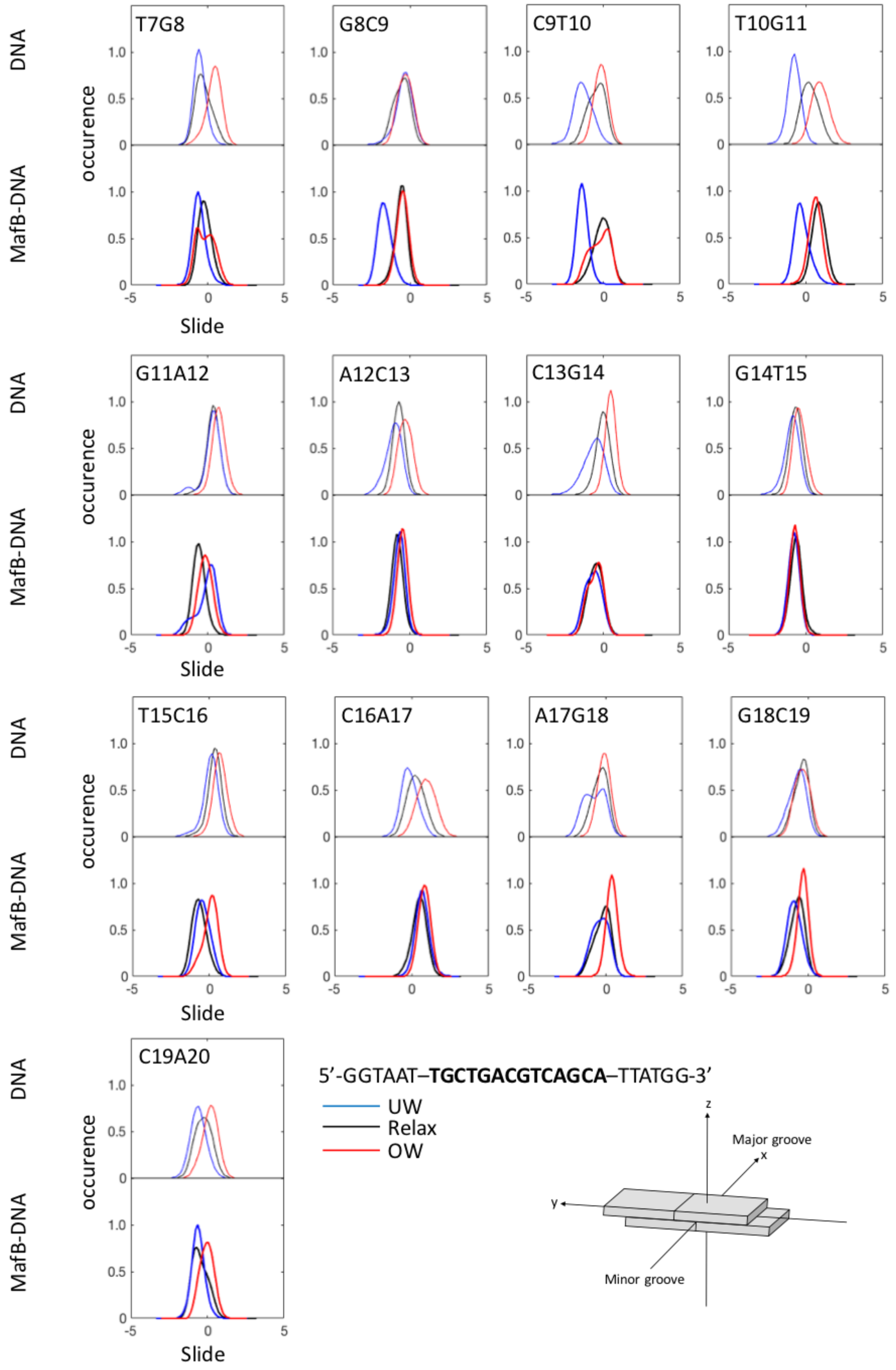

Figure S13. Slide distributions of the restrained MARE region for underwound (-4.5), relaxed and overwound (+4.5) state. MafB-DNA is denoted with thicker lines.

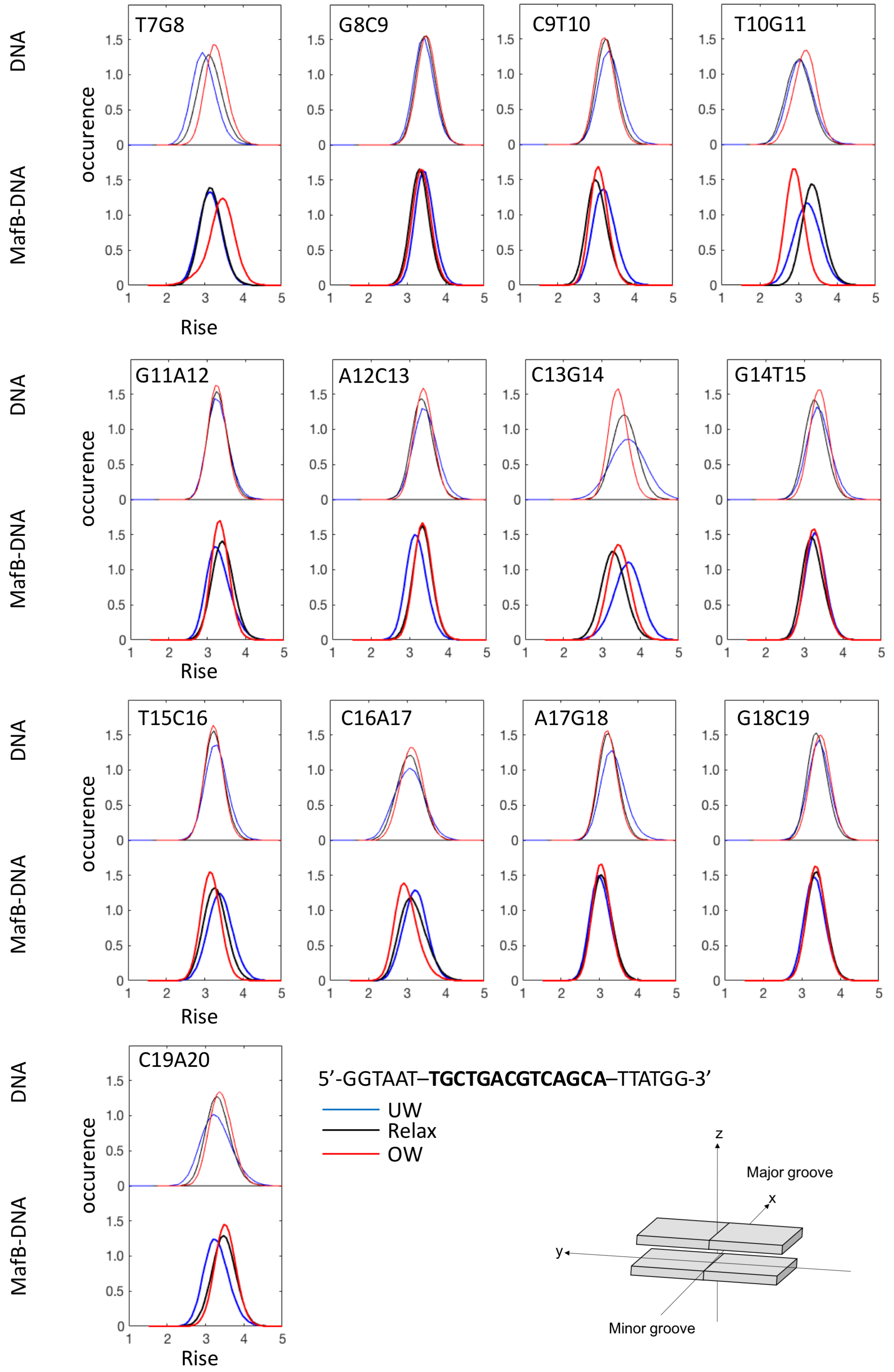

Figure S14. Rise distributions of the restrained MARE region for underwound (-4.5), relaxed and overwound (+4.5) state. MafB-DNA is denoted with thicker lines.

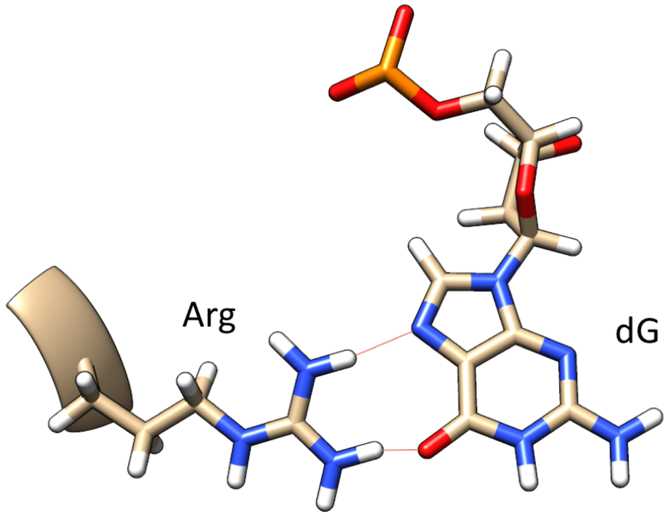

Figure S15. The strength of MafB-DNA contacts is defined by summing all the interactions formed between the residues pairs. For example, an Arg-residue that forms two hydrogen bonds with a Gua-base has a contact strength of 2.

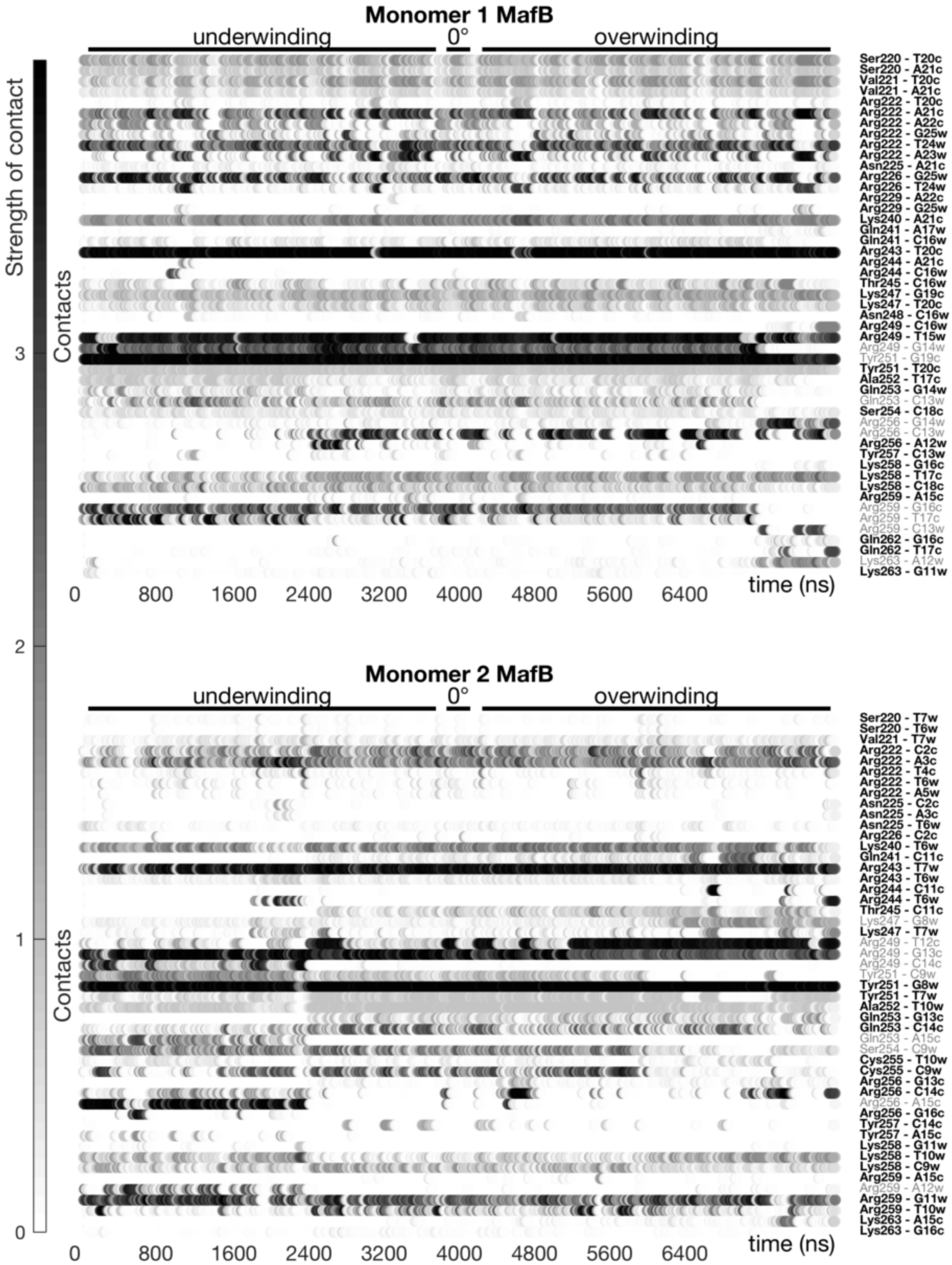

Figure S16. Dynamic interactions map illustrating non-specific MafB-DNA contacts at different degrees of torsional stress. The interactions between pairs of the protein-DNA residues are characterized by a contact strength and occurrence. Torsional stress denoted as 'underwinding' represents changes from -5 degrees/b.p. to -0.5 degrees/b.p.; and 'overwinding' – from 0.5 degrees/b.p. to 5.0 degrees/b.p. Text in bold shows contacts that change insignificantly (change in contact strength < 1) with changing torsional stress. Indices "w" and "c" indicate to Watson- and Crick-DNA strands.

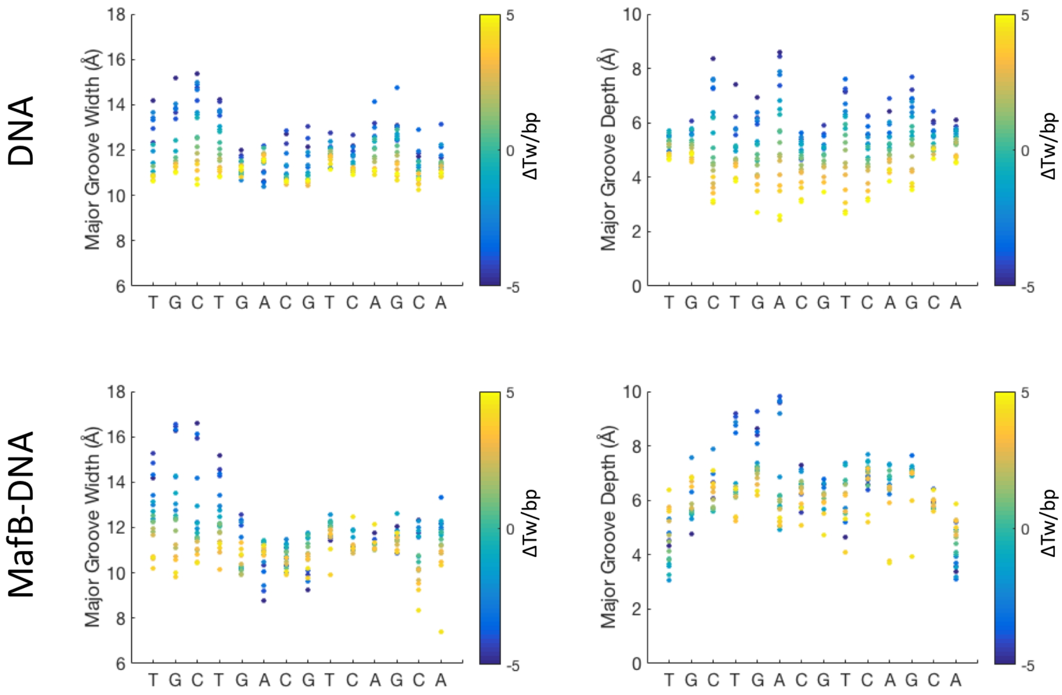

Figure S17. Major groove width and depth of the restrained MARE-region for free DNA and MafB-DNA coloured according to the imposed torsional stress.

Supplementary Videos

MafB dimer maintains contacts with torsionally stressed DNA by adjusting the structure of its DNA binding domains. At the higher degree of DNA underwinding, < -4.0°/b.p., when DNA major groove becomes wider and deeper, the long alpha-helices of MafB bend away from DNA. At the higher degree of DNA overwinding, > 4.0°/b.p., when DNA major groove becomes relatively shallow and narrow, the protein alpha-helices buckle away from DNA major groove (See supplementary movies S1 and S2).

Video S1. Conformational changes of the MafB-DNA complex as DNA experiences induced torsional stress, going from undertwisting (-4.5°) to overtwisting (+4.5°). The movie shows the complex from the “front”.

Video S2: Conformational changes of the MafB-DNA complex upon induced torsional stress, going from undertwisting (-4.5°) to overtwisting (+4.5°). The movie shows the complex from the “top”, through DNA helical axis.
